# Metalloporphyrin Zr-MOF Nanoparticles Film for Device Integration of Spin Qubits and Qudits

**DOI:** 10.1021/acs.inorgchem.6c03040

**Published:** 2026-09-21

**Authors:** Javier Serrano-Oiza, Inés Tejedor, Eva Natividad, Arnaud Hemmerle, Philippe Fontaine, Valentin Novikov, Guillem Aromí, Ignacio Gascón, Olivier Roubeau

**Affiliations:** † 16765 Instituto de Nanociencia y Materiales de Aragón (INMA), CSIC − Universidad de Zaragoza, Zaragoza 50009, Spain; ‡ Synchrotron SOLEIL, L’Orme des Merisiers, Départementale 128, Saint Aubin 91190, France; § Departament de Química Inorgànica i Orgànica, 16724 Universitat de Barcelona, Diagonal 645, Barcelona 08028, Spain; ∥ Institute of Nanoscience and Nanotechnology of the University of Barcelona (IN2UB), Barcelona 08028, Spain

## Abstract

Integration of molecular spin qubits onto superconducting circuits is one of the current proposals to build a hybrid quantum processor, in which their coupling to resonators would allow complex coherent quantum operations. As an alternative to large magnetically dilute crystals or concentrated nanoscale deposits of paramagnetic molecules studied so far, we consider here preformed nanoparticles of a Zr­(IV)-based 3D MOF doped with Cu­(II) and V­(IV)O metalloporphyrin. Highly crystalline, phase-pure nanoparticles are obtained with an adjustable number of spin carriers and a homogeneous size that is adequate for nanoconstrictions in superconducting resonators. Continuous-wave and time-domain EPR spectroscopy show that the Cu­(II) and V­(IV) spins in these particles are potential qubits and qudits, respectively, with robust quantum coherence. These nanoparticles form good dispersions in methanol-chloroform mixtures, which allows the formation of monolayers of densely packed particles at the air–water interface. These Langmuir films are successfully transferred to very different substrates, including Nb resonators, homogeneously covering their most sensitive areas, *i.e.*, the edges of the superconducting lines. Finally, the quantum coherence of the qubit nodes is maintained in the transferred nanoparticles, thereby demonstrating the viability of the present approach for the development of hybrid quantum processors.

## Graphical abstract

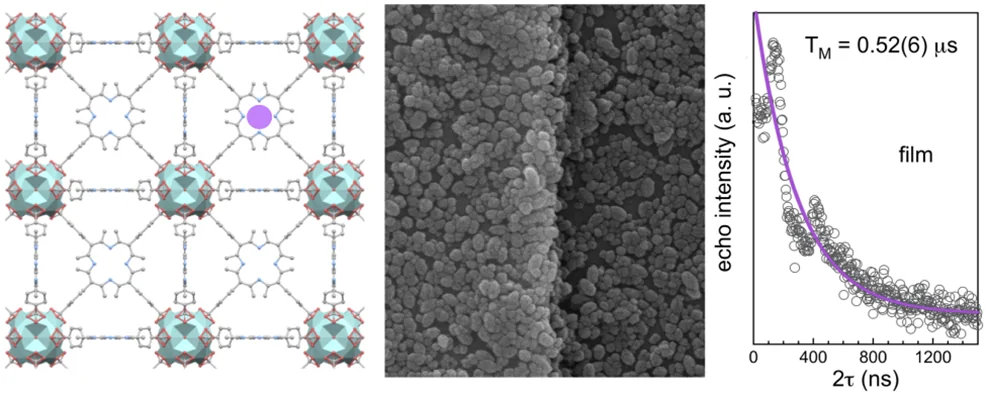

## Introduction

The anticipated advent of quantum computing,^1^ which takes advantage of fundamental principles of quantum mechanics such as superposition and entanglement to realize quantum processing of information, remains dependent on the ability to wire up and operate a sufficiently large number of qubits so as to allow the required scalability, quantum error correction, and eventually the realization of complex quantum algorithms. Even in the current NISQ (noisy intermediate-scale quantum) era,^2^ this continues to be a challenge even for the most advanced implementations of quantum hardware, i.e., superconducting qubits.^1b^ A promising emerging alternative contemplates using the electronic spins of paramagnetic molecules, either organic radicals or transition-metal ion complexes, to realize spin qubits, multiple qubits, d-dimensional qudits, and quantum gates.^3^ Synthetic control and the possibility to also use coupling with nuclear spins enable the design of multiple qubit and qudit architectures at the molecular scale, thereby allowing fine control of the topology and interqubit interactions,^3,4^ while ensuring large-scale reproducibility unmatched by any other qubit embodiments. The ability to operate quantum gate(s) or embed quantum error correction codes at the molecular scale is a differentiating advantage for facilitating the scaling of computational resources. Some theoretical proposals, as well as experimental realizations along this line, have already been reported,^5,6,7^ while chemical engineering can be used to tune qubit characteristics and isolate them from neighboring magnetic noise sources, thereby increasing their quantum coherence times.^8^ The current challenge for these so-called molecular qubits is, therefore, to wire them up into a macroscopic quantum computing architecture that would enable us to individually read and/or manipulate them,^9^ a task that is extremely difficult due to their molecular nature.

A developing proposal toward this goal couples the spin qubits to superconducting resonators and transmission lines.^9,10^ Its successful implementation would allow reading the qubits’ state in the dispersive regime, and operating qubits, qudits, or quantum gates either by inducing transitions between different spin states at a single resonator-molecular qudit, or through effective interactions among remote molecular qubits interconnected through a common superconducting line. Most experiments realized so far have involved macroscopic crystals, *i.e.*, very large spin ensembles, already demonstrating the ability to perform broadband spectroscopy as well as pulsed experiments.^11^ The ultimate goal of having one qubit/qudit per resonator relies on improving the sensitivity and design of the superconducting resonators^12^ but also necessitates methods to deposit a controlled number of qubits at nanoconstrictions made in the superconducting architecture to locally enhance the spin-photon coupling.^9,10,12b^

In doing so, a stringent requirement is that both the quantum coherence and the designed energy-level scheme encoding the qubits/qudits are preserved once implemented in the hybrid solid-state device. The flexibility and fluxionality of molecules associated with their interaction with a solid surface can indeed be counterproductive. Dip-Pen nanolithography (DPN) of nanodroplets of the organic radical DPPH recently enabled strong coupling of an ensemble of these molecular qubits to the photon(s) in the superconducting circuit.^13^ Control of orientation is nevertheless not so critical for highly isotropic radicals, and although we recently applied the same method to a [Dy_2_] complex,^14^ it is clear that the random orientation of such magnetically anisotropic molecules in the deposited droplet will complicate the use of their energy-level scheme, if not prevent it. Another strategy involves qubit monolayers formed under UHV, but it may not be applicable to the rough surfaces of the superconducting devices being developed, while the case of vanadyl phthalocyanine on a Pb single crystal is an example of how interaction with the active superconducting surface can alter the electronic properties of the deposited molecules.^15^

As an alternative to truly molecular systems, metal-organic frameworks (MOFs) built with qubit nodes offer the advantage of enforcing a homogeneous and controlled orientation through the underlying framework periodicity. 3D MOFs built on the MTCPP node (H_6_TCPP = 5,10,15,20-tetrakis­(4-carboxyphenyl)­porphine) have indeed been reported, in which the M = Cu­(II) or V­(IV)O ions serve as valid qubits.^16,17^ The case of the VOTCPP node is particularly appealing, considering that an ethyl ester analogue, [VOTCPPEt], fulfills the conditions to act as a universal 4-qubit processor or, equivalently, as a d = 16 qudit, through transitions among its electronuclear levels.^11a^ We have also reported that dilute nanodomains of 2D frameworks with metalloporphyrin qubit nodes [{MTCPP}­Zn_2_(H_2_O)_2_] can be transferred onto, or directly formed *in situ* on, the surface of superconducting lines.^18^ This strategy has made it possible to cover the surface of superconducting resonators with an adjustable number of V­(IV)O porphyrin qubits with homogeneous orientation, albeit without allowing control over nanoscale localization.^19^ For the latter, we have shown that preformed submicron particles of Y­(III) hydroxycarbonate doped with varying amounts of Gd­(III) qubits could be deposited on superconducting resonators as a few isolated particles via nanolithography, as well as in the form of compact monolayers.^20^ Here, we show that the latter strategy, used with crystalline NPs of 3D Zr-MOFs incorporating Cu­(II) and V­(IV)O porphyrin nodes, enables homogeneous coverage of superconducting resonators and maintains the quantum coherence of the qubit nodes.

## Results and Discussion

### Synthesis of Phase-Pure (M)_*x*_dPCN-224 NPs with Homogeneous Size and Adjustable Composition

From the extensive literature on porphyrin-based MOFs,^21^ we selected a series of materials based on TCPP linkers and Zr^IV^_6_O_4_(OH)_4_ cluster nodes. These differ in their topology due to differences in how they are connected through TCPP, namely 12-connected in PCN-223 ^22^ and MOF-525,^23^ 8-connected in MOF-545/PCN-222,^18,24^ PCN-225,^25^ and NU-902,^26^ and 6-connected in PCN-224,^27^ the latter being an ordered, linker-defective form of MOF-525. Its disordered analogue dPCN-224, has more recently been reported and identified as the dominant structural phase among these three related systems.^28^ One appealing aspect is that the potential effect of these different topologies on the spin relaxation of metalloporphyrin nodes could be studied. We initially targeted Cu­(II)- and V­(IV)­O-doped PCN-223 and PCN-224/MOF-525 NPs. Indeed, the conditions to obtain phase-pure NPs of both undoped PCN-223 and MOF-525 have been defined through a high-throughput approach,^29^ while the synthesis of size-controlled NPs of PCN-224 has also been reported.^30^ We first focused on the latter, indeed obtaining NPs very homogeneous in size (*ca.* 100 nm; see Figure S1). Unfortunately, these proved to be amorphous, and repetitions as well as variations of synthetic parameters did not yield crystalline particles. This is in line with the literature showing that the reproducible synthesis of this family of Zr­(IV)-TCPP MOFs is tremendously challenging,^31^ in particular that of PCN-224, which in fact seems to be elusive due to the stringent conditions required to yield its unique Zr_6_ cluster orientation and ordered TCPP linker vacancies, favoring instead its disordered analogue dPCN-224.^28^

We then turned to reproducing the reported synthesis of the smaller PCN-223 NPs and two sizes of MOF-525 NPs (Table S1).^29^ We nevertheless repeatedly found that, in our hands, and even when slightly varying the synthetic conditions, these were in fact producing either mixtures of PCN-223 and dPCN-224 phases, or phase-pure dPCN-224 (Table S1, Figures S2-S3). Importantly, we found that the dominant phase was always dPCN-224. Here it should be noted that the original work of Harris et al.^29^ predates the description of the disordered phase dPCN-224,^28^ while the diffraction data used—a 2 to 16° 2θ range—would not have allowed clear discrimination between dPCN-224 and MOF-525 anyway. It does seem now that the phase reported as MOF-525 in that work is in fact dPCN-224. Again, this is in line with the stability of this disordered form, resulting from the combination of twisted phenyl rings, coplanar carboxylate groups, and a disordered Zr_6_ cluster (see Figure 1a), which represents an energetically more favorable situation. With these observations in mind, we eventually optimized the conditions to obtain phase-pure dPCN-224 nanoparticles of *ca.* 120 nm, by using the conditions of Harris *et al.* with an increased modulator/TCPP ratio (entry 2 in Table S1, Figures S2 and S4).

**Figure 1:**
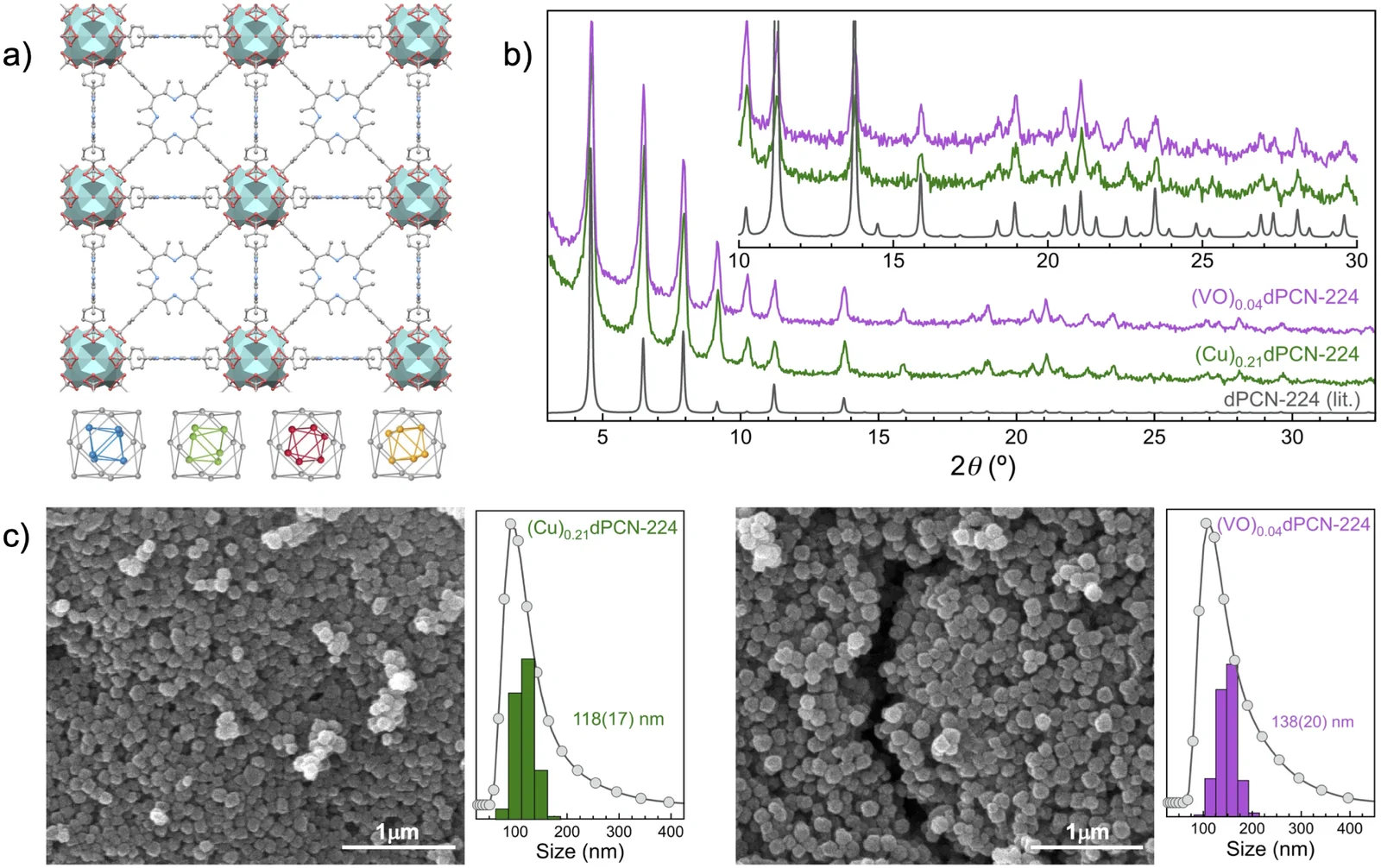
a) View of the cubic structure of dPCN-224 MOF along its *c* axis.^28^ The Zr_6_ cluster node is disordered over four orientations, shown as polyhedra. The four orientations are detailed at the bottom. b) Powder X-ray diffractograms of (Cu)_0.21_dPCN-224 (green) and (VO)_0.04_dPCN-224 (violet) compared with those calculated from the single-crystal structure of dPCN-224 (gray).^28^ c) Representative SEM images of (Cu)_0.21_dPCN-224 (left) and (VO)_0.04_dPCN-224 (right), together with the size distributions derived from analysis of SEM images (histograms and mean size) and Dynamic Light Scattering of MeOH dispersions.

We then tested the possibility of introducing varying amounts of paramagnetic MTCPP nodes (M = Cu, VO) into these NPs, while maintaining phase purity, crystallinity, and size homogeneity. In our hands, maintaining the same global porphyrin/Zr ratio and porphyrin/modulator ratio using a mixture of TCPP and MTCPP (Table S1) proved successful, as supported by structural (Figure 1b), spectroscopic (Figure S5), and microscopic (Figure 1c and S6) characterization, in addition to magnetic properties (see below).

We focus here specifically on two NP compositions, respectively, (Cu)_0.21_dPCN-224 and (VO)_0.04_dPCN-224, thus representing a wide range of paramagnetic node concentrations. Their powder diffractograms show that these are highly crystalline and indeed fully match the calculated diffractogram from single-crystal dPCN-224,^28^ in particular in the 10°–30° 2θ range where differences between MOF-525 and dPCN-224 can be spotted (Figure 1b and S2). The absence of any nanocrystallites of the ordered form PCN-224 is supported by the lack of any intensity at 2*θ* = 3.2 and 5.5°, which would correspond to its superstructure reflections. Bulk isothermal magnetization at increasing fields determined for both Cu- and VO-doped NPs fully agrees with a properly scaled Brillouin function for an *S* = 1/2 spin (Figure S7), indicating the absence of significant magnetic interactions, even in the more concentrated Cu-doped NPs. The scale factor allows the indirect determination of the doping level, yielding 3.7% and 21.3%, respectively, in excellent agreement with the composition derived from ICP-OES (SI). SEM observations show relatively monodisperse polyhedral, rather isotropic particles (Figure 1c and S6). Analysis of the particle size distribution as derived from these images indicates good size homogeneity, with mean sizes of 118(17) and 138(20) nm for (Cu)_0.21_dPCN-224 and (VO)_0.04_dPCN-224, respectively. DLS measurements on dilute acetone or methanol dispersions, readily prepared from the concentrated acetone suspension directly obtained in the synthetic procedure, fully support these observations (Figure 1c and S5). Indeed, the hydrodynamic diameter of the dispersed particles exhibits near log-normal distributions with means of 114 and 136 nm, thus in excellent agreement with sizes derived from SEM. All sizes are lower than twice the mean particle diameter, pointing to well-dispersed particles. This good size homogeneity ensures the number of spins per particle does not vary significantly from the targeted value. Repeated syntheses have shown limited batch-to-batch variations in terms of average size and doping (see Table S2). We note, however, that a fraction (1/4) of repetitions has given rise to poorly crystalline materials, with similar size and overall chemical composition (as well as similar EPR spectra; see below), for reasons not clearly understood. Such irreproducibility issues have also been observed in the bulk for this specific MOF family and have been ascribed to its very flat energy landscape and reactivity sensitive to unknown or uncontrolled factors.^28^ The fact we observe it in NPs is somewhat to be expected.

### Spin Dynamics and Quantum Coherence

Continuous-wave electron paramagnetic resonance (CW-EPR) spectra were acquired at X-band and at RT for both (Cu)_0.21_dPCN-224 and (VO)_0.04_dPCN-224 (Figure 2a).

**Figure 2:**
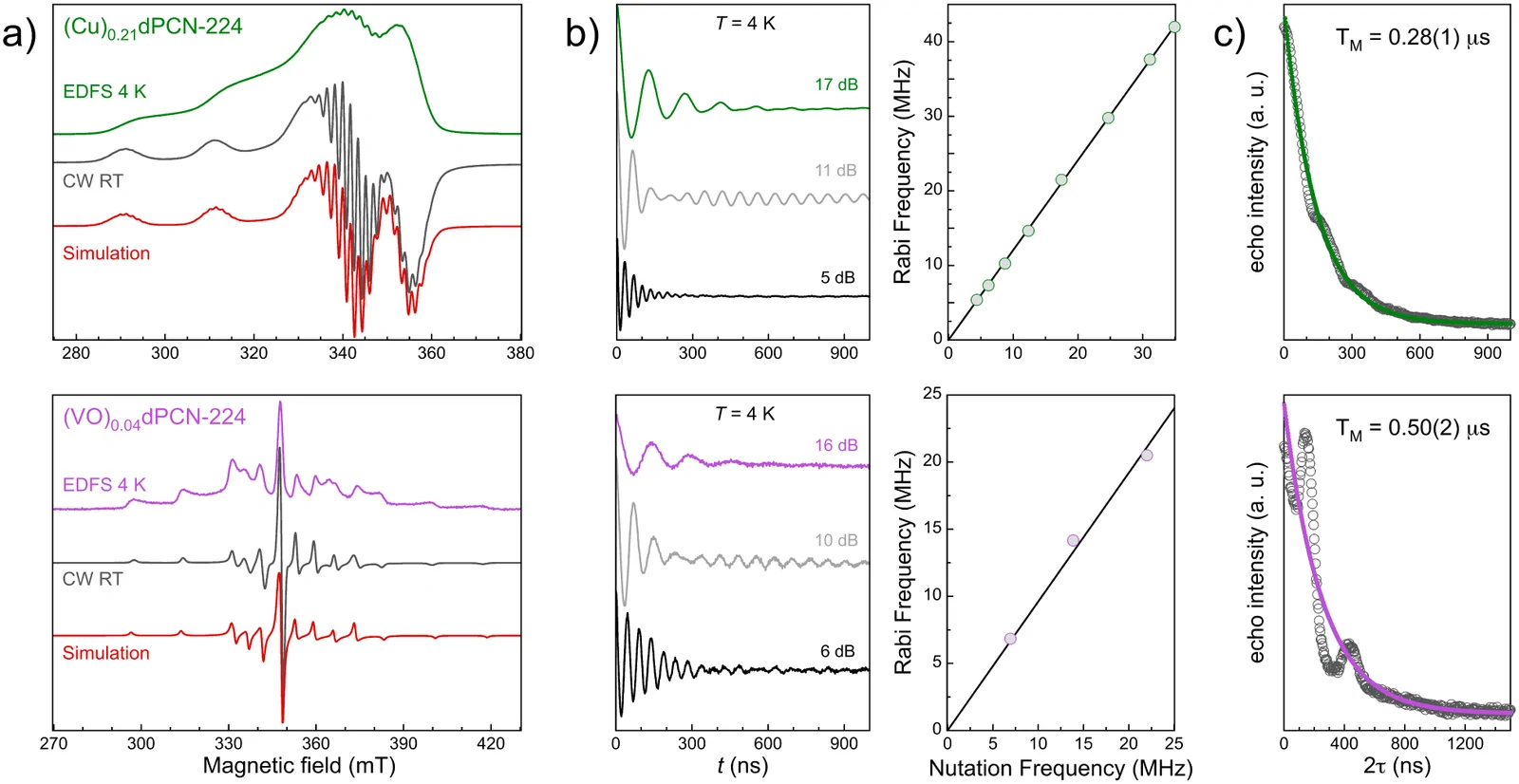
CW- and TD-EPR spectroscopy of Cu- (top) and VO-doped (bottom) dPCN-224 NPs. a) X-band CW spectrum recorded at RT and 2p-ESE-detected spectrum (τ = 200 ns) at 4 K, and simulations of the CW spectrum using an axial SH (see text). b) Rabi oscillations recorded at 4 K at 335.5 and 334.8 mT, respectively, and selected microwave power attenuations, together with the dependence of the Rabi frequency, derived from Fourier transforms of these nutation signals, on the nutation frequency. c) Hahn-echo decay at 4 K and at 310 and 334.8 mT, respectively. Colored lines are exponential decay fits used to derive the indicated phase memory times.

The former shows the characteristics of an axial Cu­(II) isolated ion, with a resolved hyperfine structure arising from both the coupling with the Cu­(II) nuclear spin and those of the coordinated N nuclei. The resolution of the hyperfine coupling confirms the absence of significant exchange interactions among the CuTCPP nodes, resulting from sufficient spatial separation, even at a relatively concentrated doping level. The spectrum can be modeled through EasySpin^32^ with the spin Hamiltonian (SH) *H* = μ_B_ **B**·**ĝ S** + **I**·**Â**·**S** where **S** is the electronic spin (*S* = 1/2) and **I** is the nuclear spin (*I* = 3/2), and **ĝ** and **Â** are, respectively, the gyromagnetic and hyperfine tensors, which have axial symmetry. A fairly good description of the spectrum is obtained with principal values g_∥_ = 2.188 and g_⊥_ = 2.068; A_∥_(Cu) = 617 MHz, A_⊥_(Cu) = 54 MHz; A_∥_(N) = 57 MHz, A_⊥_(N) = 43 MHz (Figure 2a), in line with those reported for other square-planar Cu­(II) porphyrin molecules.^18a,33^ In particular, the SH parameters are virtually identical to those reported for large crystals of the ordered Cu-PCN-224 phase,^16^ in agreement with the local Cu­(II) symmetry/environment being insensitive to structural defects. The spectrum of the VO-doped NPs is also well simulated using an axial SH including the electronic Zeeman interaction, with g_∥_ = 1.966 and g_⊥_ = 1.989, and the hyperfine interaction with the *I* = 7/2 ^51^V nucleus, with A_∥_ = 480 MHz, and A_⊥_ = 163 MHz. This is in agreement with the local symmetry at the porphyrin site, and the SH parameters are similar to those found in other vanadyl porphyrins, and in particular those in 2D and 3D MOFs.^17,18b^ Importantly, the similarity of the SH parameters of the VO-doped NPs with those of [VOTCPPEt] suggests the VO node will provide the energy-level scheme allowing one to encode a d = 16 qudit through its electronuclear transitions.^11a^ It is also worth noting that the spectra of poorly crystalline NPs remain virtually identical to those of highly crystalline ones (Figure S8), pointing to very robust energy-level schemes of the Cu­(II) and V­(IV) ions in the porphyrin coordination pocket.

Pulsed EPR was then used to evaluate the presence of quantum coherence in both Cu- and VO-doped NPs. The echo-detected field-swept (EDFS) spectra at 4 K are in excellent agreement with the RT CW-EPR spectra, as depicted in Figure 2a, confirming that the echo-detected signal over the whole range of the CW-EPR spectrum corresponds to the isolated Cu­(II) and V­(IV) ions, respectively. The EDFS spectra are, indeed, well simulated with the same SH parameters as those used for the CW spectra (Figure S9). The ability to coherently manipulate the *S* = 1/2 spins was then tested by applying a transient nutation experiment at 4 K. Rabi oscillations are thus observed for both Cu- and VO-doped NPs (Figure 2b), and the oscillation frequency derived from Fourier transforms of the nutation signal scales linearly with the applied microwave power, altogether demonstrating the viability of both metalloporphyrin nodes as quantum information carriers.

The quantum spin dynamics of these candidate qubits was estimated by determining their phase-memory time T_M_, which includes all processes leading to decoherence, including spin–spin relaxation, which is described through T_2_ and the (dipolar) coupling with nuclear spins, nuclear spin diffusion, and nuclear spin relaxation itself. Hahn-echo experiments were thus performed at 4 K and various applied magnetic fields (Figure 2c and S10). The echo decay of the Cu-doped NPs is merely monoexponential, with only a limited modulation, very similar to that of the ordered Cu-PCN-224 bulk phase at various degrees of doping.^16^ The echo decay of the VO-doped NPs shows strong modulation, while also describing an overall monoexponential trace. In the case of the Cu-doped NPs, T_M_ is found to remain relatively constant at 0.26–0.32 μs over the whole 0.1–0.5 T field range (Figure S11). The relatively short coherence is a consequence of the concentrated nature of the NPs, which increases decoherence due to spin–spin relaxation. The values of T_M_ in fact exceed the 0.16 μs reported for crystals of the ordered Cu-PCN-224 phase at a lower doping level of 10%.^16^ This favorable comparison is relatively surprising, considering that disorder can a priori only have negative effects on coherence. The VO-doped NPs exhibit longer phase-memory times, with T_M_ = 0.50 μs at the central, more intense so-called powder line, at which all orientations of the vanadyl spins are excited. The increase in T_M_ with respect to the Cu­(II) system is nevertheless not exceptional, considering the significantly more dilute character of the VO-doped NPs. It nevertheless appears to be reasonable, considering values of T_M_ reported for vanadyl porphyrin nodes in other MOFs: a longer 1.4 μs (at 6 K) in the [{VO­(TCPP)}­Zn_2_(H_2_O)_2_] 2D MOF, but at 1% doping in the ZnTCPP analogue,^18b^ and respectively 1.0 and 0.5 μs in the 3D MOF [VO­(TCPP)­Zn_2_(4,4’-bpy)] (PPF-5) at 2 and 5% doping levels in the titanyl analogue.^17^ While these can seem rather modest vs. T_M_ values reported for frozen-solutions of molecular vanadyl systems, such as [V­(C_8_S_8_)_3_]^2–^ (7 μs at 10 K),^34^ [VO­(naph-cat)_2_]^2–^ (6.5 μs at 25 K),^35^ or [VOTCPPEt] (7 μs at 25 K),^11a^ we note that the concentration of the solids mentioned above is still too high to neglect the effect of decoherence through dipolar interactions. A very recent study also reports an increase in T_M_ at 7 K for (VO)_0.05_PCN-223 solids from 0.56 μs, thus similar to what we observe here, to 0.87 μs through the inclusion of C_60_ molecules in the pores of the MOF.^36^ While the effect is ascribed to the replacement of nuclear spin-active DMF solvent molecules in the pores, there could also be an effect arising from rigidification of the lattice. In this respect, we note that [VOTCPPEt] itself has a much-reduced T_M_ in the dilute solid compared with the frozen solution,^11a^ and that this could point to the effect of lattice vibrations (as opposed to molecular ones), which in defective/disordered MOFs such as dPCN-224 could become significant.

Spin-lattice relaxation times T_1_ were thus determined for both (Cu)_0.21_dPCN-224 and (VO)_0.04_dPCN-224 NPs through the isothermal frequency dependence of their AC susceptibility under varying applied DC fields up to 1 T (Figures S12-S13). In the case of spin-1/2 systems, the derived characteristic relaxation time τ is indeed equivalent to T_1_. The field dependence of T_1_ of the Cu- and VO-doped NPs shows similar behavior, except that the former relaxes faster by about one order of magnitude (Figure 3). Thus, T_1_ increases from *ca.* 1.6/17.8 ms at 0.025 T to reach *ca.* 22/200 ms at 1 T, respectively, for the Cu/VO NPs. The increase at relatively low field to a plateau reached at 1 T likely results from the growing importance, at increased fields, of the direct relaxation process, which has a strong field dependence ≈ *B* ^–4^, contrary to spin–spin and spin–nucleus interactions, which give rise to spin relaxation by breaking the degeneracy of the Kramers doublet. The temperature dependence of T_1_ was determined for the more concentrated (Cu)_0.21_dPCN-224 up to 10 K at 0.3 T (Figure 3 right). The data are well reproduced by an a*T* ^–2^ dependence, which points to the presence of dominant phonon bottleneck effects, even though the system is not very magnetically concentrated magnetically. Possibly, this could be due to low-frequency vibrations associated with the intrinsic structural disorder of dPCN-224 acting as a temperature bath for phonon modes resonant with the spin system, and becoming the rate-determining process for the phonon relaxation time.^37^

**Figure 3:**
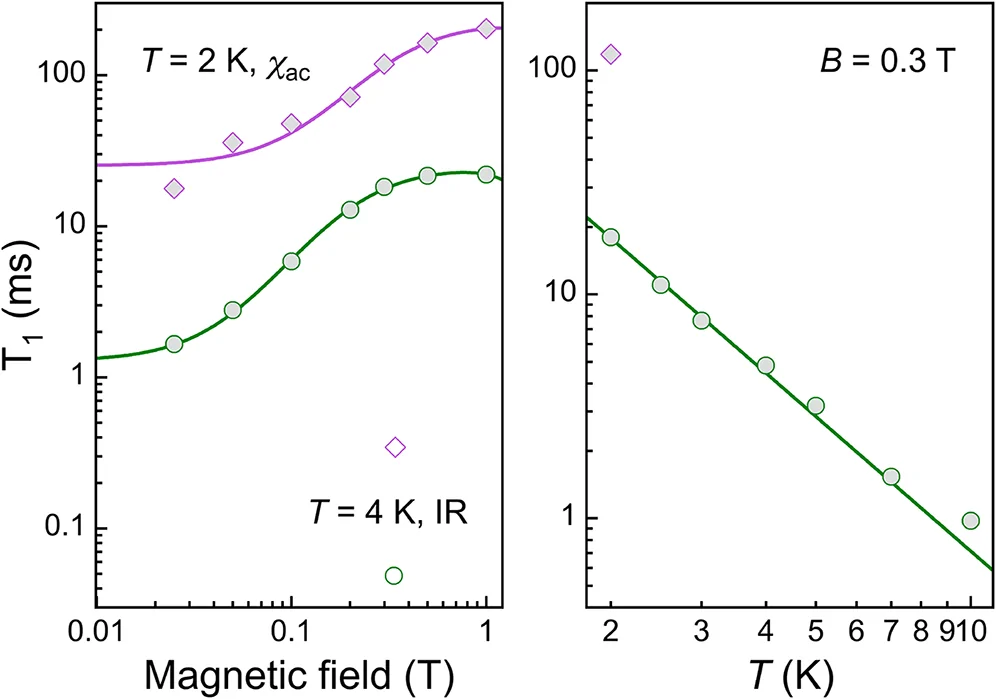
Spin–lattice relaxation time T_1_ of (Cu)_0.21_dPCN-224 (green-gray circles) and (VO)_0.04_dPCN-224 (purple-gray rhombs) NPs. Left: magnetic field dependence at *T* = 2 K. The solid lines are simulations using the Brons-van Vleck equation.^38^ Empty symbols are T_1_^fast^ determined through inversion recovery experiments at 4 K (see text). Right: temperature dependence at an applied magnetic field of 0.3 T. The full line is a fit of T_1_ = a*T* ^–2^ to the data.

We note, however, that in all cases the derived characteristic relaxation times in fact only correspond to the main relaxation mode, *i.e.*, to the main fraction of the AC susceptibility, as the real susceptibility does not reach zero at the highest measured frequency (Figures S12-S14). This means that there could be faster relaxation mode(s) at higher frequencies not accessible with our setup. Pulsed-EPR inversion recovery (IR) experiments were thus performed at short time scales. These indeed provide evidence of the presence of fast spin relaxation times, for which a good estimation is obtained by fitting a stretched exponential to the data (Figure S15), yielding mean values of T_1_^fast^ of 0.048 and 0.344 ms, respectively, *i.e.*, close to 2 orders of magnitude shorter than the main relaxation mode. These faster relaxation modes might be associated with MTCPP nodes not fully connected, *i.e.*, either in the vicinity of a Zr_6_ node defect or, more likely, not coordinated due to the disorder at the Zr_6_ node. In any case, even these T_1_^fast^ values remain more than 2 orders of magnitude larger than T_M_ and therefore do not affect the coherence times.

Altogether, our magnetically dilute dPCN-224 particles are carriers of valid spin qubits, with a number of qubits or qudits per particle that can be tuned through the level of magnetic dilution, here from 1.9 × 10^5^ Cu­(II) qubits per particle on average for (Cu)_0.21_dPCN-224 to 5.7 × 10^4^ V­(IV) qudits per particle on average for (VO)_0.04_dPCN-224. Going to much lower numbers is *a priori* feasible, on the one hand by further dilution and, on the other hand, by reducing the size of NPs using higher modulator/TCPP ratios. The present 120–140 nm diameter range is indeed a maximum if one is to use nanoconstrictions.^9,10,12,13,18b^

### Formation and Transfer of NP Monolayers

For the integration of these qubit/qudit carriers, we turned to Langmuir techniques. Indeed, transfer of dense Langmuir films of MOF NPs has proven to be very effective at homogeneously covering large-area devices, even when these present relatively large variations in height, such as interdigitated electrodes^39^ or superconducting lumped-element resonators.^19^ To use Langmuir techniques, the ability to form sufficiently stable and concentrated dispersions of the NPs in volatile organic solvents, ideally CHCl_3_ or CH_2_Cl_2_, is key. Dispersion of our dPCN-224 NPs in these solvents, however, proved impossible (Figure S4). Meanwhile, acetone, in which good concentrated dispersions are readily obtained from the synthetic procedure, is not an ideal dispersant because of its relatively high miscibility with water. Unfortunately, mixtures of these with CHCl_3_ resulted in a rapid destabilization of the dispersion with visual flocculation (Figure S16). Eventually, optimal conditions were redispersing NPs isolated from the acetone dispersions in MeOH, without drying (*i.e.*, directly from the centrifuge), followed by a 1:3 dilution with CHCl_3_ for a final concentration of 2.5 mg/mL (Figure 4a and S16). In the optimization process, we found that prolonged exposure of the NPs to MeOH results in amorphization of an initially crystalline material (Figure S17), despite the recognized stability of Zr­(IV)-based MOFs. It is therefore key that the dilution is done rapidly to minimize the time that the NPs are dispersed in MeOH. DLS of the MeOH:CHCl_3_ dispersions shows distributions close to log-normal, centered at 119 and 139 nm, respectively, for (Cu)_0.21_dPCN-224 and (VO)_0.04_dPCN-224, virtually unchanged from pure MeOH dispersions and in good agreement with sizes derived from SEM. Repeated measurements over time show that the dispersions are stable, and the fact that all sizes are below twice the average hydrodynamic diameter confirms the dispersion of isolated NPs.

**Figure 4:**
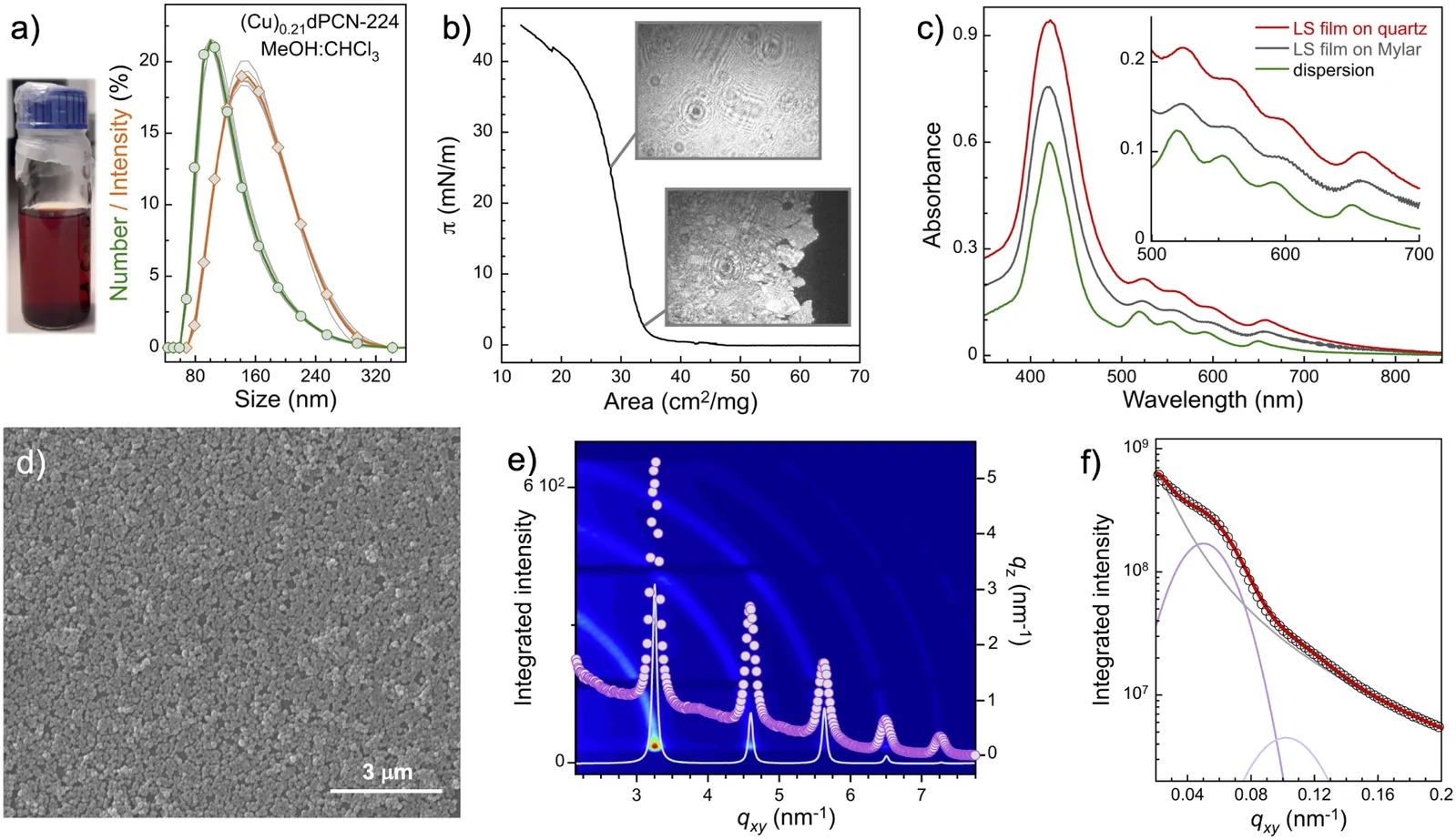
a) Picture of a 1:3 MeOH:CHCl_3_ dispersion of (Cu)_0.21_dPCN-224 NPs together with its Dynamic Light Scattering intensity and number data. Grey lines are consecutive measurements, while thicker colored lines are averages of all 6 measurements. b) Representative surface-pressure isotherm obtained for (Cu)_0.21_dPCN-224 NPs from MeOH:CHCl_3_ dispersions. BAM images (*ca.* 5 × 4 mm^2^) taken at *ca.* 2 and 25 mN/m are included, showing, respectively, a large domain of NP film and full coverage of the air-water interface. c) UV–Vis spectra of (Cu)_0.21_dPCN-224 NPs films transferred on quartz and Mylar by reverse-Langmuir-Schaefer, compared with that of the used MeOH:CHCl_3_ dispersion (2.5 mg/mL, the absorbance multiplied by 0.663 for clarity). d) Representative SEM image of a (VO)_0.04_dPCN-224 NP film transferred on Si by inverse-Langmuir-Schaefer. e) GIXD patterns of the same film (green-gray symbols), compared with the patterns calculated from the structure of dPCN 224.^28^ These I­(*q*) data are derived from the full 2D plot shown as background, by integrating radially. f) GISAXS data for the same film. The thick red line is the best fit of the data by a multiple power-law background (main component *q* ^–2.40^, gray line) and two Gaussian peak(s) with maxima at *q* = 0.050(2)/0.102(3) nm^–1^ and widths of 0.024(2)/0.030(3) nm^–1^ (purple lines).

After expanding the optimized MeOH:CHCl_3_ dispersions on water in a Langmuir trough, compression results in reproducible π-A isotherms that are very similar for both (Cu)_0.21_dPCN-224 and (VO)_0.04_dPCN-224. These show a steep increase in surface pressure to values above 40 mN/m (Figure 4b), indicative of the formation of dense and rigid Langmuir films.^40^ Brewster angle microscopy images obtained during film formation show the presence of large domains already at low surface pressure and full coverage of the water surface above *ca.* 5 mN/m.

Similar images, *albeit* with increasing intensities, are obtained upon compression, indicating densification but, *a priori*, no significant modifications of the film packing. Vertical transfer of the formed NPs film onto a Si substrate was first attempted at 25 mN/m using the Langmuir–Blodgett technique, in which the substrate is initially immersed in the subphase before film formation and then lifted. SEM observations show only partially and unevenly coated substrates (Figure S19). No improvements were obtained upon transferring at higher pressures, using lower or higher vertical speeds, or by vertically immersing the substrate from the air into the subphase through the MOF film and then lifting it. Most likely, the cohesion among the NPs in the Langmuir film is not sufficient once the water interface is replaced by the Si substrate. Horizontal transfers, *i.e.*, Langmuir-Schaefer (LS) and reverse-LS transfers, were therefore attempted and allowed efficient transfer of dense films. The UV–vis spectra of films transferred onto Mylar and quartz are basically identical to that of the dispersion, except for the slightly broader nature of both the Soret peak at *ca*. 420 nm and Q-bands (Figure 4c), as expected for a solid deposit. Although transferred under the same conditions, the film on quartz has *ca*. 20% higher absorbance, pointing to a denser coating than on Mylar. Together with surface elemental analysis (Figures S20-S21), this supports the conclusion that the chemical nature of the NPs is not affected by the transfer process. SEM observations of the films transferred on Si substrates clearly show complete coatings of NPs monolayers (Figure 4d and S22), with the reverse-LS conditions providing the best homogeneity and density of the films.

Grazing incidence X-ray diffraction (GIXD) measurements of films transferred to Si substrates demonstrate that the crystallinity of the NPs is not lost throughout the whole process of Langmuir film formation, transfer, and washing/drying. Indeed, a 1D plot of the *q*_*z*_-integrated intensity at low *q*_*xy*_ perfectly matches the diffractogram calculated from the reported dPCN-224 crystal structure (Figure 4e and S23). The full 2D GIXD plot exhibits rings corresponding to each *hkl* reflection, thereby pointing to randomly oriented NPs in the monolayer. Grazing-incidence small-angle X-ray scattering (GISAXS) patterns were also acquired to ascertain potential (local) organization of the NPs in the film. The 2D plots for both (Cu)_0.21_dPCN-224 and (VO)_0.04_dPCN-224 hint at the presence of one broad (symmetric) maximum that can *a priori* be ascribed to Bragg rods resulting from particle–particle distances in the films (Figure S24). Examination of the integrated *q*_*z*_ intensity versus *q*_*xy*_ data confirms the presence of one clear peak, and hints at the presence of a second, more faint one (Figure 4f and S25). The data can indeed be satisfactorily reproduced using two Gaussian peaks on top of a power-law background, yielding peak maxima of 0.050(2)/0.050(1) and 0.100(3)/0.102(3) nm^–1^, respectively for (Cu)_0.21_dPCN-224 and (VO)_0.04_dPCN-224 films. Assuming a close-to-hexagonal packing, the first peak would correspond to a center-to-center distance *D* = 4π/*q√*3 of 145 nm, in good agreement with particle sizes derived from SEM and DLS (see above). The large width of this main peak, at 0.025(2)/0.024(2) nm^–1^ however points at a very limited correlation length of the supposed hexagonal packing of roughly 300 nm, *i.e.*, 2–3 particles. This is in agreement with the position of the second peak which is at about twice the first one, thus corresponding to a primitive arrangement, as opposed to the 11 peak of an hexagonal packing that would be expected at √3 times the first peak. Therefore, despite their relatively good size homogeneity, the NPs only form very small, locally packed, organized domains, which is indeed in agreement with SEM observations. Nevertheless, homogeneous monolayers of Cu- and VO-doped NPs of the dPCN-224 MOF can be deposited on very different substrates.

### Device Integration and Quantum Coherence

The ability to integrate NPs with a controlled number of qubit/qudit nodes on a functional device was tested using Nb superconducting resonators, given the relative chemical similarity of Nb’s outermost oxide layer surface to that of a Si substrate. Thus, the same optimal conditions as for Si substrates were used to transfer (Cu)_0.21_dPCN-224 and (VO)_0.04_dPCN-224 NP Langmuir monolayers onto, respectively, a chip with various Lumped Element Resonators (LERs)^19^ and another chip with a coplanar waveguide resonator.^11a^ Thanks to their flexible design, LERs are currently used for state-of-the-art studies toward the optimal coupling of molecular spin qubits.^9,41^ SEM observations show in both cases that the film homogeneously covers the whole chip surface (Figure 5a, S26-S27), despite the *ca.* 0.5 mm height of the Nb lines and the resonators’ complex structure. Importantly, the particles fully cover both the lower Si surface and the higher Nb surface up to their edges (Figure 5b,c, S26-S27), the latter being the most sensitive areas of the superconducting lines, as the local magnetic field there is maximal.^11,13^

**Figure 5:**
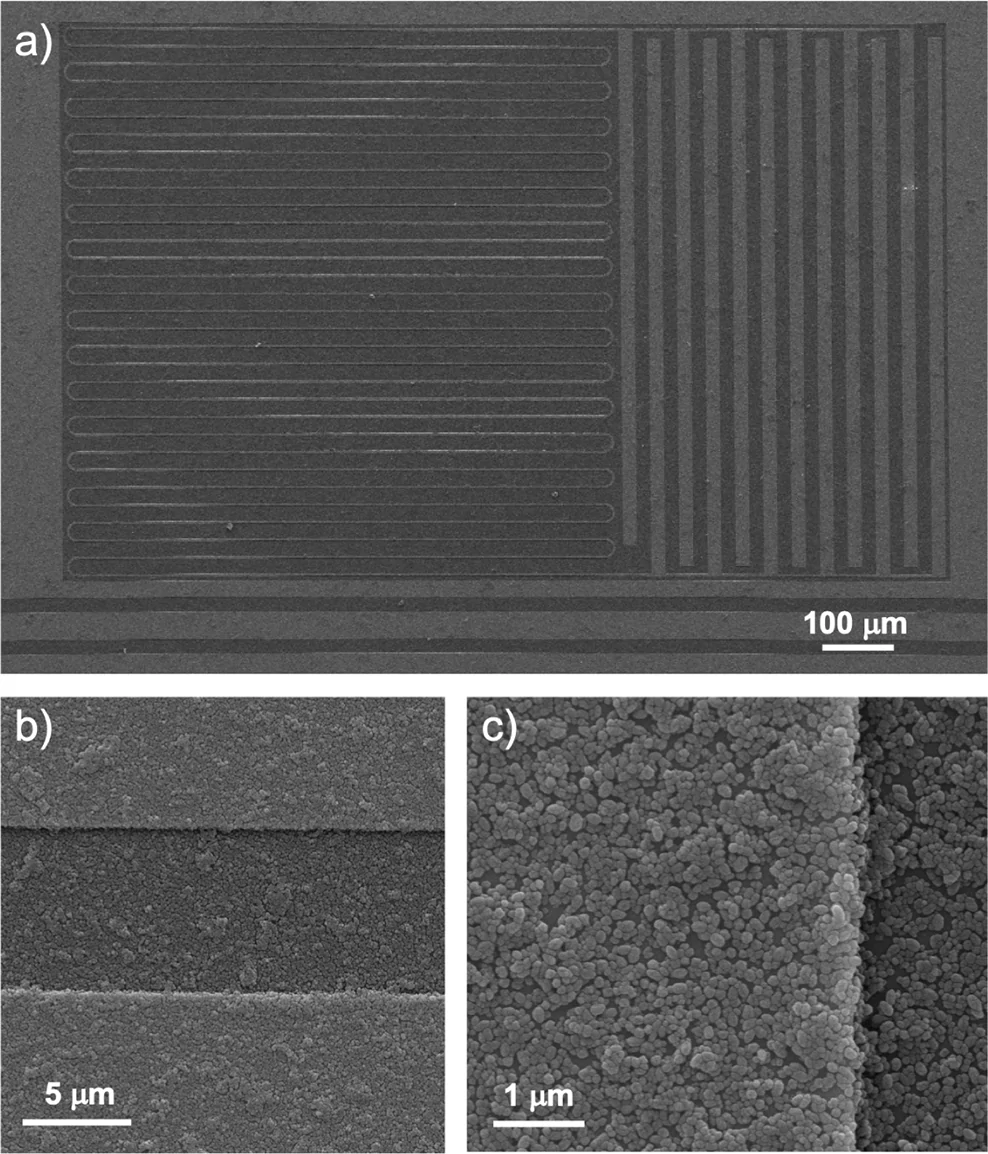
a) Representative SEM image of (Cu)_0.21_dPCN-224 NPs film transferred onto a chip with superconducting Nb LERs at 25 mN/m. b) and c) Higher-magnification images of horizontal and vertical superconducting lines of the resonator, showing full coverage to their edges.

A critical aspect of using molecular spin qubits is that their energy level schemes are maintained once they are integrated into a solid-state device, and similarly important is whether their quantum coherence is (un-)­affected by this integration. We therefore studied the (Cu)_0.21_dPCN-224 and (VO)_0.04_dPCN-224 films with pulsed EPR, using Mylar as a substrate to allow slicing a relatively large area into pieces that are then packed in parallel in the EPR tube. EDFS spectra were successfully obtained at 4 K, showing excellent agreement with those of the bulk solids (Figure 6 left), taking into account the higher noise level associated with the low number of spins. This means that the energy level schemes, determined by the spin Hamiltonian parameters that allow simulation of both CW and EDFS spectra, remain the same for NPs in the monolayer films. Further, the echo decays of both films, determined through Hahn-echo experiments at 4 K, are also virtually identical to those obtained for the bulk solid. This logically results in very similar T_M_ values of 0.29(3) and 0.52(6) μs (Figure 6 right), thereby demonstrating that the quantum coherence of the Cu­(II) and V­(IV)­O qubit nodes is unaffected by the integration process used in this work. Clearly, this is likely thanks to the use of MOF NPs that remain crystalline, thereby ensuring that the framework acts as protection against any modification of the qubit environment. Here, it should be pointed out that reports on the quantum coherence of molecular spin qubits in thin films are extremely scarce, and are limited to molecules diluted in a diamagnetic matrix.^42^ To a large extent, this is due to the necessity of isolating spin qubits, resulting in an unfavorable combination of spin density and sensitivity, even motivating the specific design of a Fabry-Pérot resonator to facilitate the measurement of thin-film samples of paramagnetic species such as molecular quantum bits.^43^

**Figure 6:**
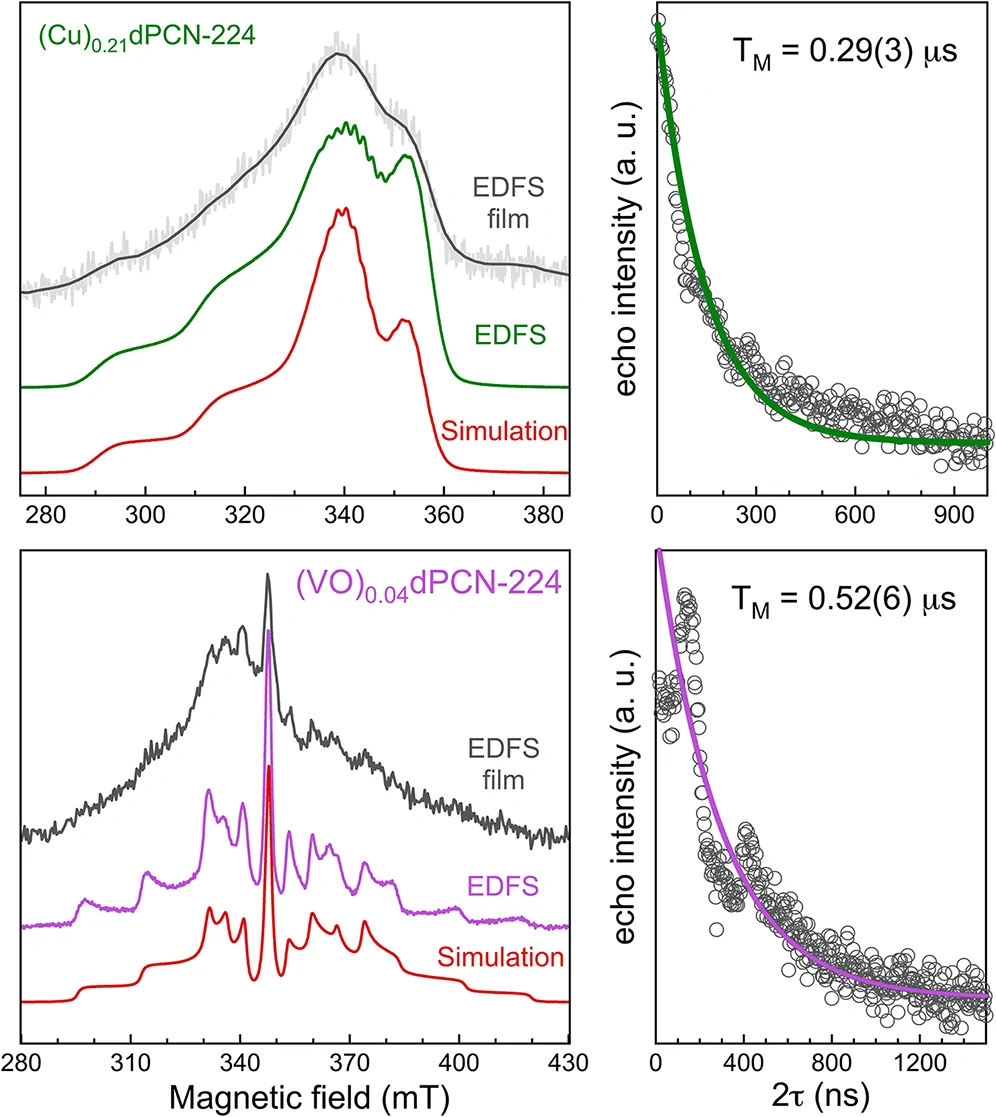
TD-EPR spectroscopy of Cu-doped (top) and VO-doped (bottom) dPCN-224 NP monolayers on Mylar. Left: 2p-ESE-detected field-swept spectrum (τ = 200 ns) at 4 K, compared with that of the bulk NPs, and simulations using the same axial SH as for CW spectra (see text). The EDFS spectrum in the Cu case is shown as a light gray line, and, being particularly noisy, a smoothed spectrum is plotted on top as dark gray line for clarity. Right: Hahn-echo decay at 4 K and at 310 and 334.8 mT, respectively. Colored lines are exponential decay fits used to derive the indicated phase memory times.

## Conclusions

Highly crystalline, phase-pure nanoparticles of the 3D Zr­(IV)-based MOF dPCN-224 doped with variable amounts of CuTCPP or VOTCPP linkers can be obtained with homogeneous sizes in the 110–140 nm range. The metalloporphyrin centers show the typical axial spin Hamiltonian parameters observed in molecular analogues, which, in the case of the vanadyl system, ensure that the energy-level scheme allows encoding a d = 16 qudit. The Cu­(II) and V­(IV)O spins in these NPs maintain significant quantum coherence, with phase-memory times T_M_ of up to *ca.* 0.3 and 0.5 μs, respectively, values that are reasonable considering the relatively low degree of dilution of the NPs (*ca.* 21% and 4%, respectively). The ability to coherently manipulate these spin qubits is demonstrated by Rabi oscillations observed in nutation experiments.

The (M)_*x*_dPCN-224 NPs form stable dispersions in acetone, MeOH, and MeOH:CHCl_3_, with the latter allowing the use of Langmuir–Blodgett techniques for their integration into solid-state devices. Using reverse Langmuir-Schaefer transfers of films formed at the air-water interface, densely packed NPs monolayers are successfully transferred onto very different substrates; this strategy therefore provides the means to integrate ensembles of an adjustable number of spin qubits and qudits onto, *a priori,* any device. This allowed us to homogeneously cover superconducting resonators, including the edges of the Nb lines, thereby favoring maximized spin-photon coupling. The demonstration that both the energy-level scheme and quantum coherence of the qubit nodes in the NPs are maintained in these monolayers validates the use of preformed NPs of regular frameworks for the safe integration of spin qubit nodes into solid-state devices. On the way toward developing a viable hybrid quantum computing architecture, control of the orientation and localization of these NPs nevertheless remains necessary. We are currently studying the use of AFM-based lithography with the current NPs dispersed in long-chain alcohols and surface pre-functionalization for this.

## Experimental Section

### Chemicals

5,10,15,20-tetrakis­(4-carboxyphenyl)­porphine (H_6_TCPP, >97%) was purchased from TCI. VO­(SO_4_)·H_2_O (>99.0%), Cu­(CH_3_COO)_2_·H_2_O (>99.0%), ZrOCl_2_·8H_2_O (>99.0%), benzoic acid (99%), glacial acetic acid, and reagent- and HPLC-grade solvents, N,N’-dimethylformamide (DMF), chloroform, acetone, and methanol were purchased from Sigma-Aldrich and used without further purification.

Nb superconducting resonators were fabricated as described previously.^11a,19^

### Synthesis

[Cu­(H_4_TCPP)] was obtained as a DMF solvate from H_6_TCPP and copper acetate in DMF, as described previously.^18a^ [VO­(H_4_TCPP)] was synthesized by hydrolysis of crystals of the tetraethyl ester analogue [VO­(TCPPEt)],^11a^ as described previously.^18b^ ICP-OES and magnetization versus field measurements at low temperatures agree with a composition of [VO­(H_4_TCPP)]_0.32_[H_6_TCPP]_0.68_.

*Synthesis of Cu*_0.21_ *-dPCN-224 and VO*_0.04_ *-dPCN-224 NPs*: Adapting conditions from Harris *et al.*,^29^ ZrOCl_2_·8H_2_O (5.2 mg, 0.0016 mmol) and a mixture of H_6_TCPP (8.81 mg, 0.011 mmol) and [M­(H_4_TCPP)] (0.99 mg, 0.001 mmol) were dissolved in 3 mL of DMF with bath ultrasonication (10 min) in a 20 mL borosilicate vial. Acetic acid (0.35 mL) was then added, and the resultant solution was heated in the closed vial for 18 h in an oven at 90 °C. The nanoparticles were collected by centrifugation, followed by washing with DMF (2 mL × 3 times) and acetone (2 mL × 3 times).

### Langmuir and Langmuir–Blodgett Film Fabrication

Surface pressure–area (π–A) isotherms were obtained using a Teflon Langmuir trough (NIMA model 702; dimensions 720 mm × 100 mm). Langmuir–Blodgett and Langmuir–Schaefer films were made with a KSV-NIMA trough, model KN 2003, with dimensions of 580 mm × 145 mm. Brewster angle microscopy (BAM) images were acquired using a KSV NIMA Micro BAM, equipped with a red laser (659 nm, 50 mW). More details are given in the Supporting Information

### Physical characterization

*Powder X-ray diffraction* data were obtained at ambient temperature using a Rigaku D-Max diffractometer equipped with a rotating anode and a graphite monochromator to select the Cu Kα wavelength, at the Servicio General de Apoyo a la Investigación (SAI) of the Universidad de Zaragoza.

*Infrared spectra* were acquired of neat samples using a PerkinElmer Spectrum 100 apparatus equipped with an ATR device. *UV–vis spectra* were acquired with a Varian Cary 50 spectrophotometer.

*Field-emission scanning electron microscopy* observations were made through the Laboratorio de Microscopías Avanzadas (LMA) with an FEI INSPECT-F50 microscope working at 10 kV. Samples were coated with a conductive layer of Pd.

*Surface elemental analysis* of the films was performed using a JEOL JXA-iHP200F Field Emission Electron Probe Microanalyzer at the Servicio General de Apoyo a la Investigación (SAI), Universidad de Zaragoza.

*Magnetic measurements*were performed with a Quantum Design MPMS3 magnetometer equipped with a SQUID sensor, hosted by the Physical Measurements Unit of the Servicio General de Apoyo a la Investigación (SAI), Universidad de Zaragoza. Isothermal alternating-current (AC) measurements were collected in the range 0.4 ≤ *ν* ≤ 900 Hz with a 4 Oe amplitude.

*Electron Paramagnetic Resonance*(EPR) experiments, both continuous-wave (CW) and pulsed time-domain (TD), were carried out using a Bruker Elexsys E580 spectrometer operating in X-band, equipped with a Stinger cryogen-free variable-temperature control system, a Flexline cryostat, and an ER 4118X-MD5 dielectric resonator. Hahn echo decays were recorded using the sequence: *(π/2)–d*_1_ *– (π)–d*_1_ *–echo*, with *d*_1_ incremented in 2-ns steps starting from 100 ns. Pulse lengths were 8 ns for π/2 and 16 ns for π. The total envelope of the spin echo was integrated. Inversion recovery measurements employed a conventional three-pulse sequence: *(π)–d*_1_ *– (π/2)–d*_2_ *–(π)–d*_2_ *–echo*. The pulse lengths were 8 ns for π/2 and 16 ns for π; the inter-pulse delay d_2_ was set to 400 ns, with an initial inversion delay *d*_1_ of 400 ns. Rabi oscillations were recorded by applying a nutation microwave pulse of variable length, adding a constant delay of 10 ms, much longer than T_M_ time, and monitoring the echo intensity as a function of nutation pulse duration. The nutation pulse width was incremented in steps of 2 ns. Baseline correction (first-order polynomial), an exponential windowing function, zero-padding, and Fourier transformation were applied to the time-domain signal. The resulting spectra were analyzed to extract Rabi frequencies. The dependence of the experimental Rabi frequency on the calculated nutation frequency (assuming 44 MHz at 0 dB attenuation) was evaluated to verify the expected microwave power-scaling behavior. Simulations of the spectra were obtained with the EasySpin program.^32^

*Dynamic light scattering* (DLS) experiments were conducted using a Malvern Zetasizer Nano ZS ZEN3600 equipped with a 633-nm laser and configured in a backscattering arrangement at 173°. Measurements were performed at 25 °C at concentrations on the order of 2.5 mg/mL or lower.

*Grazing-incidence X-ray diffraction* (GIXD) and *grazing-incidence small-angle X-ray scattering* (GISAXS) studies were performed at SIRIUS Beamline of the SOLEIL Synchrotron (Saint Aubin, France).^44^ The measurements were performed using an incident X-ray beam of 8 keV (λ = 0.155 nm), a beam size of 0.1 × 0.5 mm^2^ (V × H), and an incident angle of 2.0 mrad. GISAXS measurements were performed using a 2D PILATUS3 1M (Dectris, Switzerland) detector located 4.5 m from the sample. A vertical tungsten rod is used as a beam stop for the direct and reflected beams. A flight-path tube flushed with helium is located between the sample and the detector in order to reduce absorption and scattering by air. The GIXD setup uses a 2D PILATUS3 1M (Dectris, Switzerland) detector combined with a Soller collimator of 0.075° resolution to scan the in-plane 2θ angle while measuring, at each 2θ point, the vertical distribution of the scattered intensity.

## Supplementary Material



## Notes

Further synthetic and experimental details : SEM observations, UV-Vis spectra, pressure-area isotherms, PXRD, DLS, magnetization, AC susceptibility, CW and pulsed EPR, surface microanalysis, GIXD and GISAXS data, as described in the text (PDF)
