# Supplementary material for: Metalloporphyrin Zr-MOF Nanoparticles Film for Device Integration of Spin Qubits and Qudits
Source: Inorg Chem. 2026 Sep 21;65(39):23092–102. doi: 10.1021/acs.inorgchem.6c03040 (PMC13644307; doi:10.1021/acs.inorgchem.6c03040)
Supplement: Supplementary file 1 [file ic6c03040_si_001.pdf]

Supporting information for the manuscript

Metalloporphyrin Zr-MOF nanoparticles film for device integration of spin qubits and qudits

Javier Serrano-Oiza,[a] Inés Tejedor,[a] Eva Natividad,[a] Arnaud Hemmerle,[b] Philippe Fontaine,[b] Valentin Novikov,[c,d] Guillem Aromí,[c,d] Ignacio Gascón[a] and Olivier Roubeau[a,*]

a Instituto de Nanociencia y Materiales de Aragón (INMA), CSIC – Universidad de Zaragoza, 50009, Zaragoza, Spain. E-mail: roubeau@unizar.es
b Synchrotron SOLEIL, L'Orme des Merisiers, Gif-sur-Yvette 91192, France
c Departament de Química Inorgànica i Orgànica, Universitat de Barcelona, Diagonal 645, 08028 Barcelona, Spain
d Institute of Nanoscience and Nanotechnology of the University of Barcelona (IN2UB), Barcelona, Spain

Table of contents

**Synthetic details** incl. **Tables S1** and **S2**	p. S2
**Figure S1.** Representative SEM and PXRD of NPs : PCN224 procedure	p. S4
**Figure S2.** Representative SEM and PXRD of NPs : PCN223/MOF525 procedure	p. S7
**Figure S3.** PXRD of NPs from modifications of PCN223/MOF525 procedure	p. S8
**Figure S4.** SEM of dPCN-224 NPs and DLS of dispersions in acetone, MeOH and $CHCl_3$	p. S9
**Figure S5.** UV-Vis spectra of dPCN-224, $(Cu)_{0.21}$dPCN-224 and $(VO)_{0.04}$dPCN-224 NPs	p. S10
**Figure S6.** Additional SEM of $(Cu)_{0.21}$dPCN-224 and $(VO)_{0.04}$dPCN-224 NPs	p. S11
**Figure S7.** Magnetization at 2 K of $(Cu)_{0.21}$dPCN-224 and $(VO)_{0.04}$dPCN-224 NPs	p. S12
**Figure S8.** Comparison of cw-EPR of highly crystalline and poorly crystalline NPs	p. S13
**Figure S9.** EDFS spectra of $(Cu)_{0.21}$dPCN-224 and $(VO)_{0.04}$dPCN-224 and simulation	p. S14
**Figure S10.** 2p Hahn-echo decays at 4 K and variable fields	p. S15
**Figure S11.** Field dependence of phase memory times T_M at 4 K	p. S16
**Figure S12.** Frequency dependence of ac susceptibility at 2 K and variable fields (Cu)	p. S17
**Figure S13.** Frequency dependence of ac susceptibility at 2 K and variable fields (VO)	p. S18
**Figure S14.** Frequency dependence of ac susceptibility at 0.3T and variable *T* (Cu)	p. S19
**Figure S15.** Inversion Recovery decays at 4 K	p. S20
**Table S3.** Parameters describing the 2p ESE decays, ac susceptibility and IR data	p. S21
**Figure S16.** Appearance and DLS of MeOH:$CHCl_3$ and acetone:$CHCl_3$ dispersions	p. S22
**Figure S17.** PXRD of $(Cu)_{0.21}$dPCN-224 upon prolonged dispersion in MeOH	p. S23
**Langmuir and Langmuir−Blodgett film fabrication**	p. S24
**Figure S18.** π−A isotherm and material accumulation with not-ideal dispersions	p. S25
**Figure S19.** Representative SEM observations of LB transfers	p. S26
**Figure S20.** Surface microanalysis of $(Cu)_{0.21}$dPCN-224 NPs film on Si	p. S27
**Figure S21.** Surface microanalysis of $(VO)_{0.04}$dPCN-224 NPs film on Si	p. S28
**Figure S22.** Additional SEM images of a reverse LS film on Si wafer at 25 mN/m	p. S29
**Figure S23.** GIXD 1D patterns and 2D plot of $(Cu)_{0.21}$dPCN-224 NPs reverse-LS film	p. S30
**Figure S24.** GISAXS 2D data for $(Cu)_{0.21}$dPCN-224 and $(VO)_{0.04}$dPCN-224 NPs films	p. S31
**Figure S25.** GISAXS 1D data for $(Cu)_{0.21}$dPCN-224 NPs film	p. S32
**Figure S26.** SEM images of $(Cu)_{0.21}$dPCN-224 NPs film on a LER	p. S33
**Figure S27.** SEM images of $(VO)_{0.04}$dPCN-224 NPs film on a CPW resonator	p. S34

Reagents and precursors

Commercial 5,10,15,20-tetrakis(4-carboxyphenyl)porphine (H_6TCPP, >97%) was purchased from TCI. $VO(SO_4)\cdot H_2O$ (>99.0%), $Cu(CH_3COO)_2\cdot H_2O$ (>99.0%), $ZrOCl_2\cdot 8H_2O$ (>99.0%), benzoic acid (99%), glacial acetic acid and reagents/HPCL grade solvents N,N'-dimethylformamide (DMF), chloroform, acetone and methanol were purchased from Aldrich and used without further purification.

[Cu(H_4TCPP)] was synthesized from H_6TCPP and copper acetate in DMF as a DMF solvate as described previously.[1]

[VO(H_4TCPP)] was synthesized by hydrolysis of crystals of the tetra-ethyl-ester analogue [VO(TCPPEt)],[2] as described previously.[3] ICP-AES and magnetization vs. field at low temperatures however indicate that a significant portion of the porphyrin is unmetallated, overall agreeing with a composition of $[VO(H_4TCPP)]_{0.32}[H_6TCPP]_{0.68}$. This batch was used anyway as the aim is to obtain MOF NPs doped with a fraction of metalated porphyrin nodes. We note however that further repetitions of the synthesis always resulted in a fraction of unmetallated porphyrin, *albeit* varying from few up to 70%, possibly due to de-metallation during hydrolysis of the ester groups.

Synthesis of MOF nanoparticles

<u>PCN224</u>

Following the work of Zhou et al. reporting on the size-controlled preparation of NPs of PCN-224,[4] 300 mg ZrOCl2·8H2O, 50 mg H6TCPP and 7 mL benzoic acid were mixed in 100 mL DMF in a 250 mL round-bottom flask and heated at 90ºC under stirring for 5h. After cooling and standing to RT to allow the formed solid to deposit at the bottom of the flask, the supernatant DMF was removed. 30 mL fresh DMF were then added to the solid and the suspension was centrifugated, and washed one more time with DMF and two times with 30 mL MeOH.

An alternative synthesis was attempted repeating the procedure above but adding 1 mL H_2O to reaction mixture, following literature that points at the amount of trace water in DMF as a key parameter to successfully form the PCN-224 phase.[5] by decantation and the solid was recovered by adding 30 mL of fresh DMF.

Neither synthesis nor repetitions gave the targeted PCN-224 phase (Fig. S1), and this method was thus abandoned.

[1] A. Urtizberea, E. Natividad, P. J. Alonso, M. A. Andrés, I. Gascón, M. Goldmann, O. Roubeau, *Adv. Funct. Mater.*, **2018**, 28, 1801695

[2] I. Gimeno, A. Urtizberea, J. Román-Roche, D. Zueco, A. Camón, P. J. Alonso, O. Roubeau, F. Luis, *Chem. Sci.*, **2021**, 12, 5621-5630

[3] A. Urtizberea, E. Natividad, P. J. Alonso, L. Pérez-Martínez, M. A. Andrés, I. Gascón, I. Gimeno, F. Luis, O. Roubeau, *Mater. Horiz.*, **2020**, 7, 885-897

[4] J. Park, Q. Jiang, D. Feng, L. Mao, H-C. Zhou, *J. Am. Chem. Soc.*, **2016**, 138, 3518-3525

[5] C. Koschnick, M. W. Terban, S. Canossa, M. Etter, R. E. Dinnebier, B. V. Lotsch, *Adv. Mater.*, **2024**, 36, 2210613

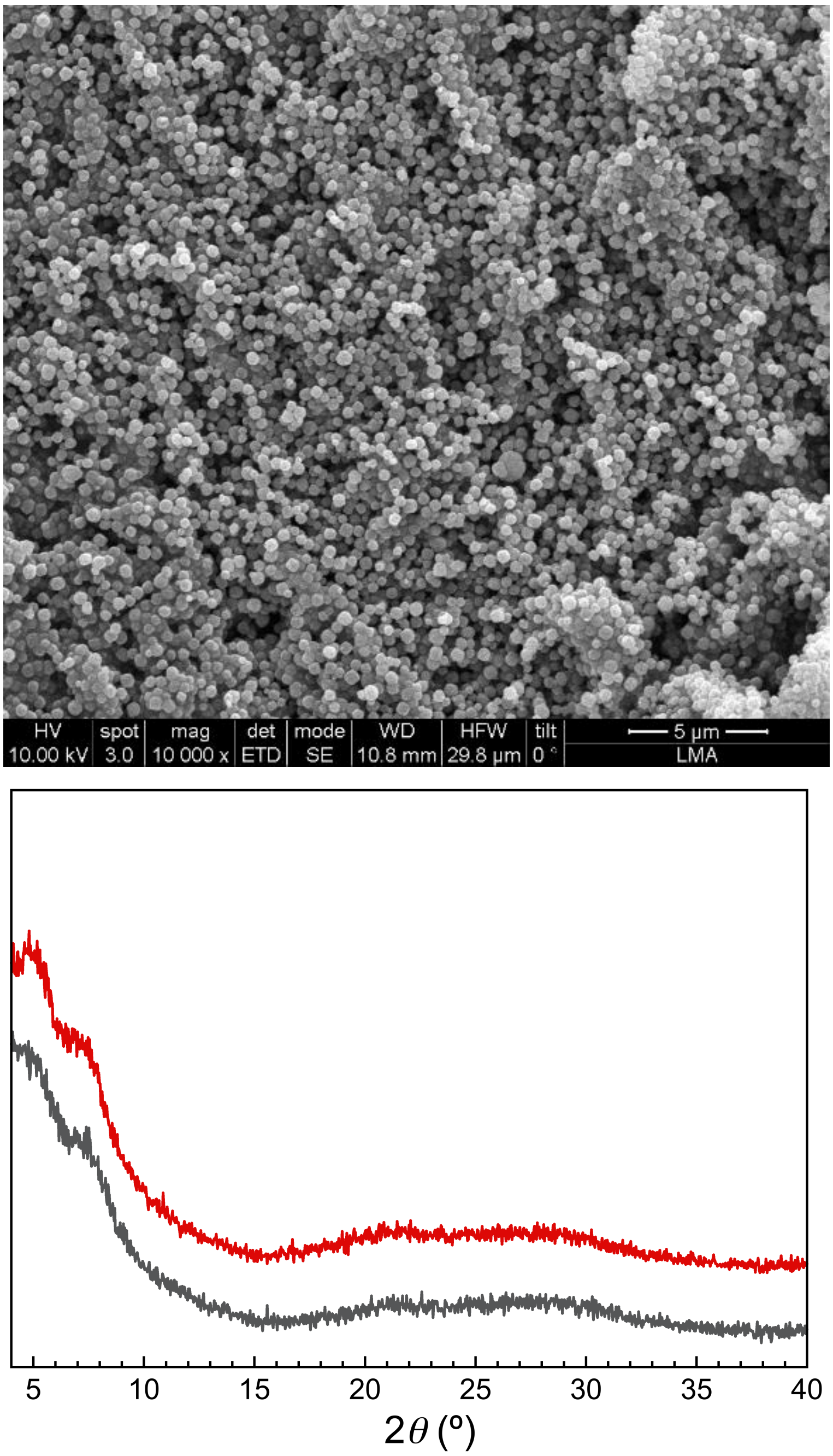

Figure S1. Top: representative SEM image of NPs obtained following the reported procedure for PCN-224 NPs.[5] Bottom: typical powder X-Ray diffraction patterns obtained for the synthesis done either without (red trace) or with (grey trace) adding water to the reaction mixture (see above).

PCN223 and MOF525, dPCN-224 and $(M)_x$dPCN-224

The synthetic conditions used are based on the high-throughput study of Harris et al.[6] It first involves the dispersion of $ZrOCl_2 \cdot 8H_2O$, H_6TCPP in DMF with bath ultrasonication (10 minutes) in a 20 mL borosilicate vial. Acetic acid is then added and the resultant solution is heated in the closed vial during 18 hours in an oven at 90 ºC. The nanoparticles are collected by centrifugation followed by washing with DMF (2mL × 3 times) and acetone (2 mL × 3 times). The proportions of the different reagents, acetic acid as well as the concentration are the parameters optimized in the original work of Harris et al.[7] Table S1 details the exact conditions used in this work for representative samples.
We first targeted the synthesis of NPs of MOF-525 with 2 sizes, 122 and 341 nm, (entries 1 and 2 in Table S1) and the smaller PCN-223 NPs (538x331 nm, entry 3 in Table S1), using the optimized conditions originally reported by Harris et al.[6] Note that these were done multiple times with similar results in terms of size and phase-purity.

Considering that the conditions for entry 2 gave a phase-pure sample of seemingly MOF-525, but in fact dPCN-224, while those of entry 1 and 3 were giving a mixture of PCN-223 and dPCN-224 (Fig. S2), we attempted to obtain phase-pure PCN-223 (entries 4-7), focusing in particular on changing the relative amount of the acetic acid modulator. Increasing it however favored the dPCN-224 phase, while decreasing it resulted in amorphous solids.

We eventually decided to continue the study using dPCN-224 NPs. The conditions of entry 2 were therefore repeated several times to ensure they give reproducible results. One out of four repetitions gave a poorly crystalline sample, without clear experimental reason.

The synthesis of $(M)_x$dPCN-224 NPs (M=Cu and VO) was then done using these conditions and maintaining the same global porphyrin/Zr ratio and porphyrin/modulator ratio using a mixture of TCPP and MTCPP. In our hands, x could be varied from *ca.* 0.01 to 0.25 without altering significantly the average size of the obtained NPs, while maintaining the phase purity. We observed that while the VO content was in line with the used reagent ratio (taking into account the $[VO(H_4TCPP)]_{0.32}[H_6TCPP]_{0.68}$ composition, see above), the Cu content was systematically larger in the obtained NPs with respect to the used reagent ratio. The details of the two batches eventually used for EPR spectroscopy and transfer to Si substrate, namely $(Cu)_{0.21}$dPCN-224 and $(VO)_{0.04}$dPCN-224, are given in Table S1 (entries 8 and 9). The formulations are based on ICP-OES which resulted in Cu/Zr and V/Zr ratios of 0.14 and 0.027 respectively. These are in good agreement with the estimation derived from *M vs. H* data at low temperature (see Fig. S7). Table S2 gathers information on reproducibility tests of these same conditions, as well as variations of the level of doping.

[6] M. L. Kelty, W. Morris, A. T. Gallagher, J. S. Anderson, K. A. Brown, C. A. Mirkin, T. D. Harris, *Chem. Commun.*, **2016**, 52, 7854-7857

Table S1. Representative synthetic conditions used and outcome based on powder X-ray diffraction results. Those highlighted in colours are those studied further in this work.

entry	$ZrOCl_2{\cdot}8H_2O$ (mg)	H_6TCPP (mg)	$M(H_4TCPP)$ (mg)	DMF (mL)	Acetic acid (mL)	Crystallinity	Phase(s)
1	5.20	9.80	-	3	0.2	Poor	PCN-223 +dPCN-224
2	5.20	9.80	-	3	0.35	Good	dPCN-224
3	5.20	9.80	-	3	0.4	Intermediate	PCN-223 +dPCN-224
4	10.39	19.63	-	6	0.8	Good	dPCN224
5	10.40	19.60	-	6	0.9	Good	dPCN-224 + traces PCN-223
6	10.39	19.61	-	6	1.0	Good	dPCN-224 + traces PCN-223
7	10.42	19.60	-	6	0.6	Poor	?
8	5.19	8.81	0.99 (M=Cu)	3	0.35	Good	dPCN-224
9	5.19	8.81	0.99 (M=VO)*	3	0.35	Good	dPCN-224

* as mentioned above, the composition is in fact $[VO(H_4TCPP)]_{0.32}[H_6TCPP]_{0.68}$

Table S2. Representative synthetic conditions used for reproducibility testing and variation of the metal composition, x for M_xdPCN-224 (M=Cu in green or VO in violet).

entry	$ZrOCl_2 \cdot 8H_2O$ (mg)	H_6TCPP (mg)	$M(H_4TCPP)$ (mg)	DMF (mL)	Acetic acid (mL)	Crystallinity	Doping (x)
8	5.19	8.81	0.99 (M=Cu)	3	0.35	Good	0.21[a]
8b	5.20	8.94	0.98 (M=Cu)	3	0.35	Good	0.19[b]
8c	5.19	8.98	1.01 (M=Cu)	3	0.35	Poor	0.21[b]
8d	5.21	8.83	0.99 (M=Cu)	3	0.35	Good	0.20[b]
9	5.19	8.81	0.99 (M=VO)*	3	0.35	Good	0.04[a]
9b	5.20	8.60	1.05 (M=VO)*	3	0.35	Poor	0.05[b]
9c	5.20	8.80	1.00 (M=VO)*	3	0.35	Good	0.04[b]
9d	5.19	8.85	0.97 (M=VO)*	3	0.35	Good	0.04[b]
10	5.29	9.72	0.18 (M=Cu)	3	0.35	Good	0.04[b]
11	5.23	9.13	0.42 (M=Cu)	3	0.35	Poor	0.10[b]
12	5.12	6.43	2.65 (M=VO)*	3	0.35	Intermediate	0.11[b]
13	5.13	8.75	0.42 (M=VO)*	3	0.35	Good	0.02[b]

* as mentioned above, the composition is in fact $[VO(H_4TCPP)]_{0.32}[H_6TCPP]_{0.68}$; a: determined through both ICP metal analysis and isothermal magnetization at 2 or 5 K; b: determined through isothermal magnetization at 2 or 5 K

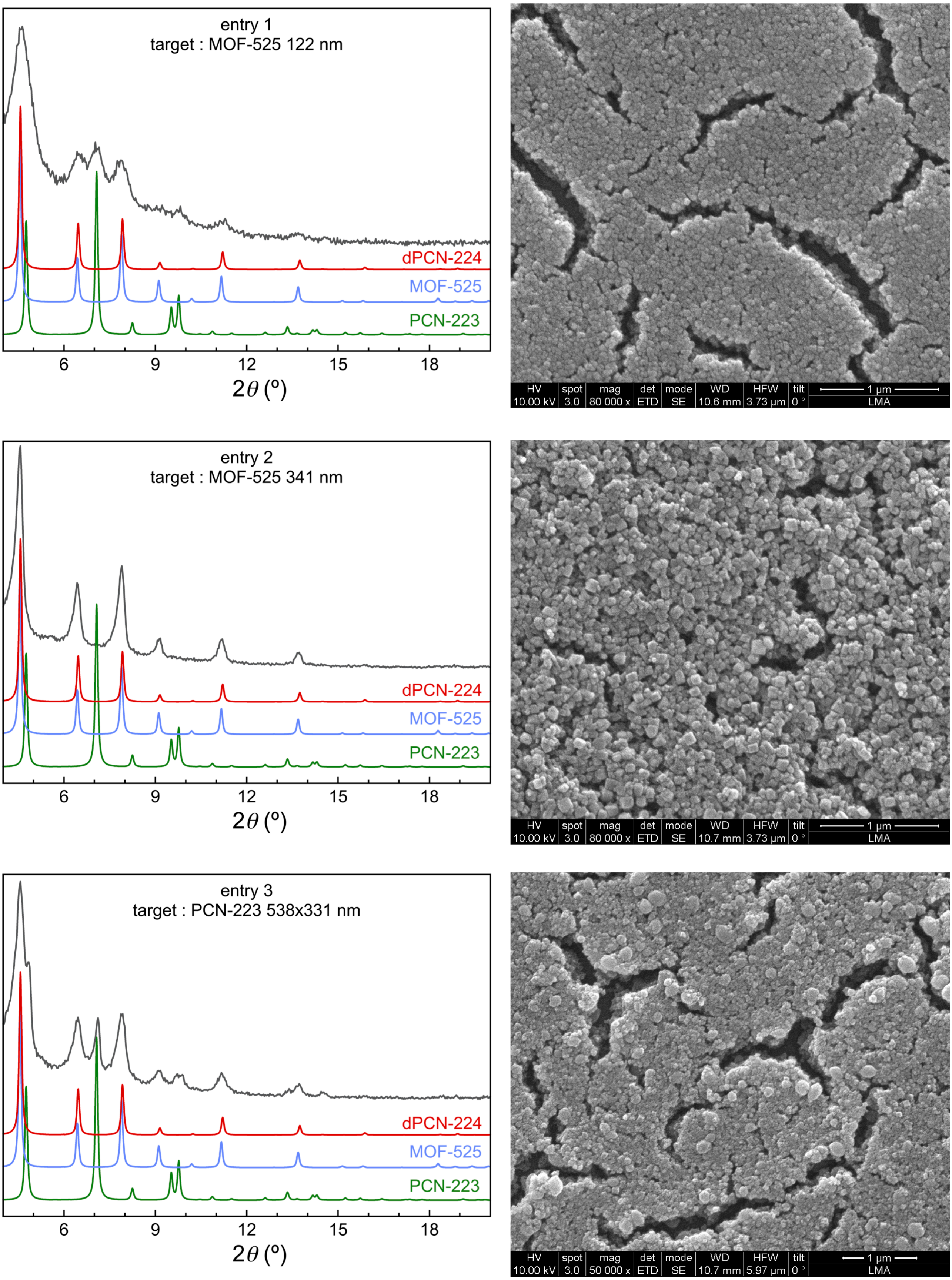

Figure S2. Powder X-Ray diffraction patterns (left) and SEM observations (right) for entries 1, 2 and 3 (see Table S1). The calculated PXRD patterns of MOF-525, PCN-223 and dPCN-224 are obtained using the reported single crystal structures.

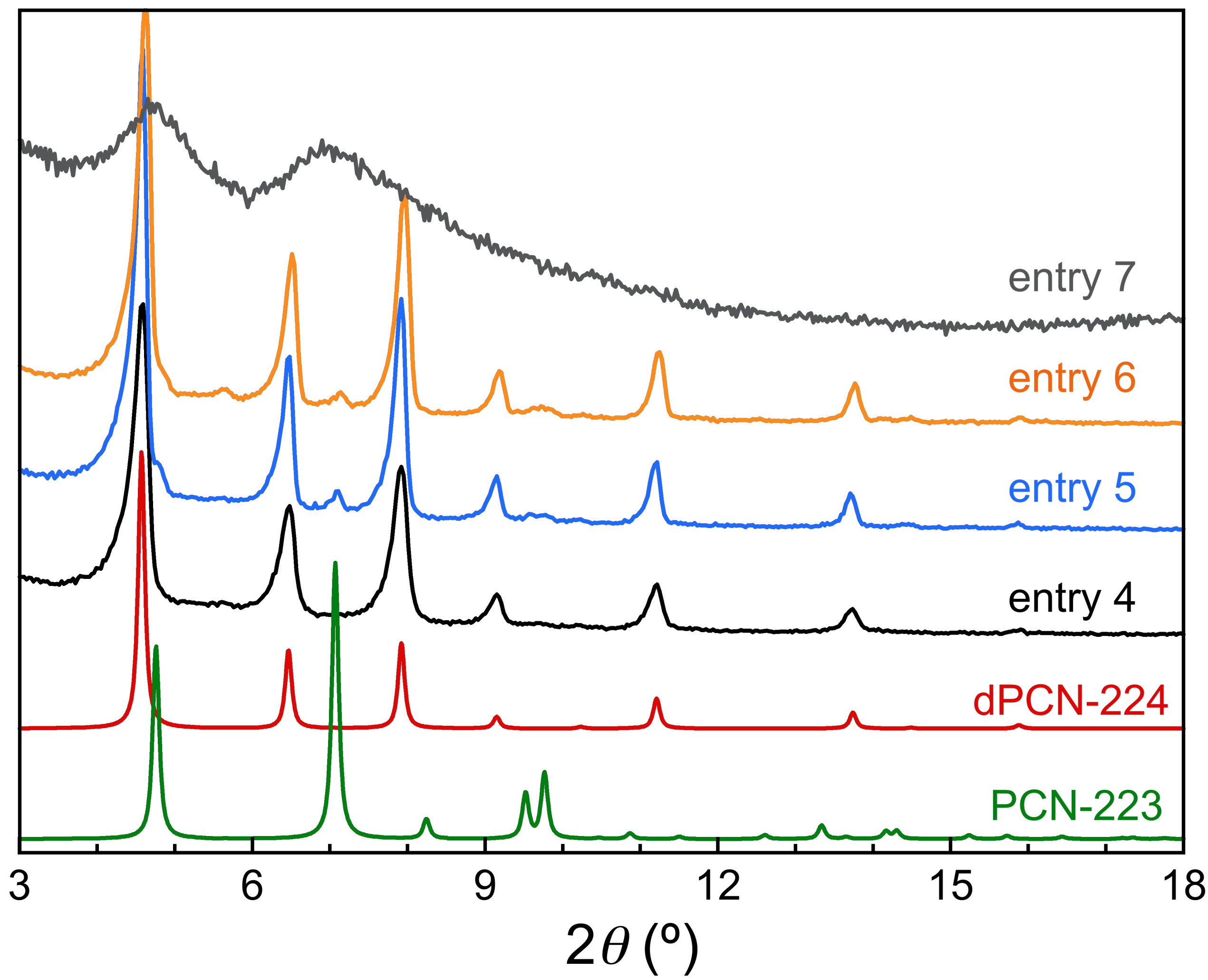

Figure S3. Powder X-Ray diffraction patterns for entries 4, 5, 6 and 7 (see Table S1), showing in the first three either pure dPCN-224 phase or mixture of phase with dominant dPCN-224 and minor traces of PCN-223. The calculated PXRD patterns of PCN-223 and dPCN-224 are obtained using the reported single crystal structures.

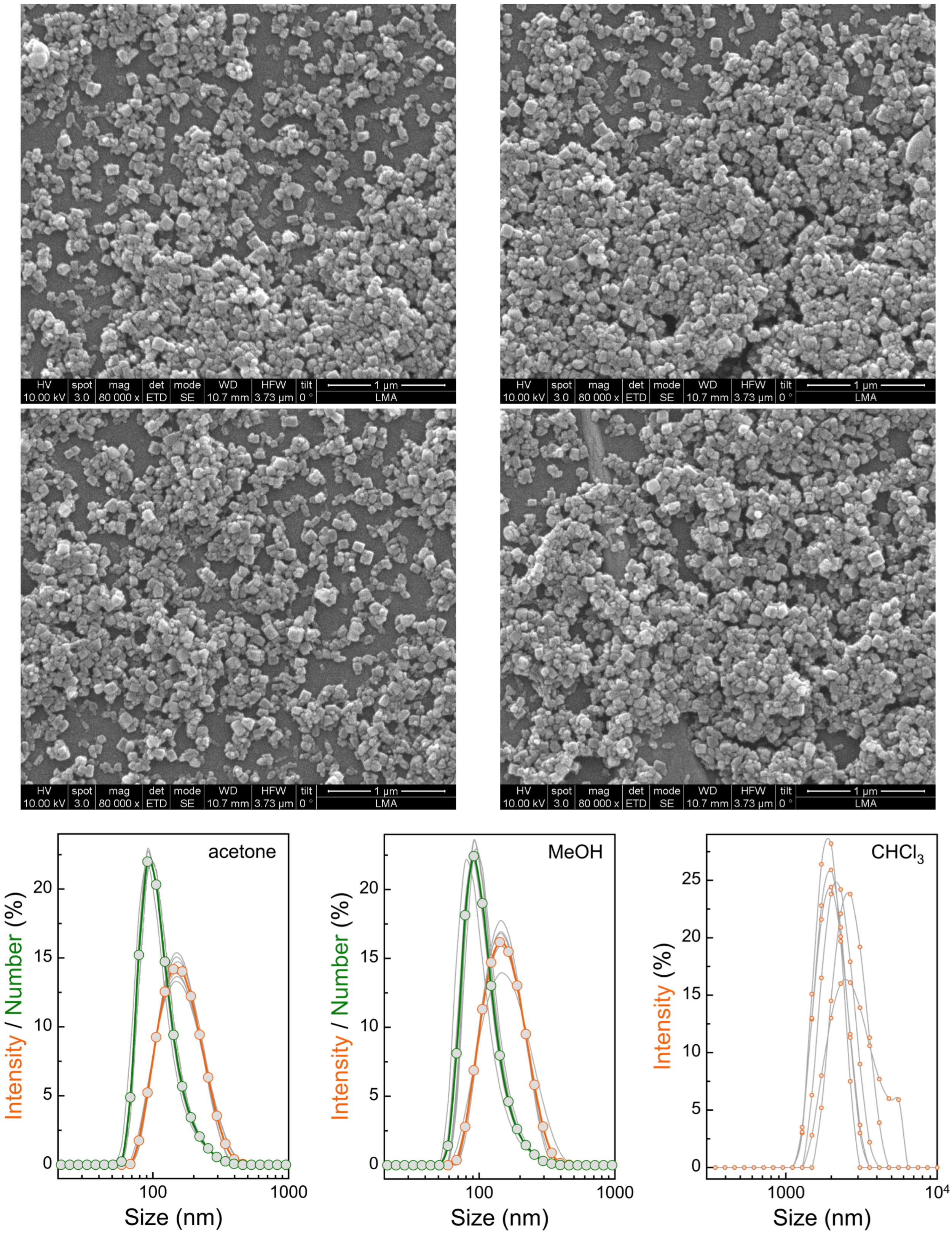

Figure S4. Top: additional SEM images of dPCN-224 NPs obtained along the conditions of entry 2 in Table S1. Bottom: Dynamic Light Scattering for dispersions of the same NPs in acetone, MeOH and $CHCl_3$, as indicated. Grey lines are successive measurements, while thicker green and orange lines are averages of these.

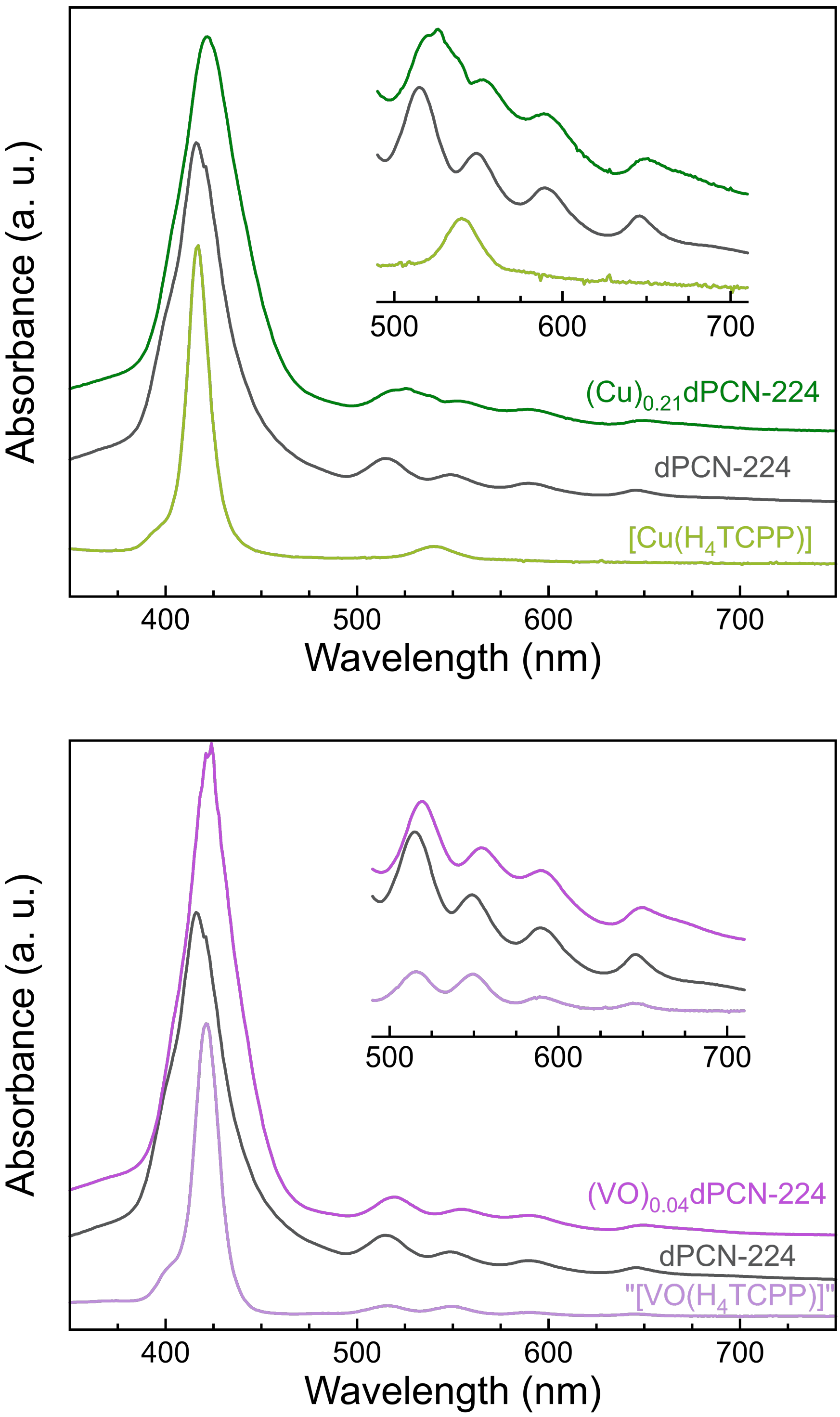

Figure S5. UV-Vis spectra of $(Cu)_{0.21}$dPCN-224 (top, entry 8 in Table S1) and $(VO)_{0.04}$dPCN-224 (bottom, entry 9 in Table S1) dispersions compared with those of a dPCN-224 NPs dispersion (entry 2 in Table S1, grey lines) and of solutions of the precursors [Cu(H_4TCPP)] and [VO(H_4TCPP)]$_{0.32}$[H_6TCPP]$_{0.68}$. The insets show enlarge views of the Q-bands range. Dispersions are in 1:3 MeOH:$CHCl_3$.

(Cu)$_{0.21}$dPCN-224

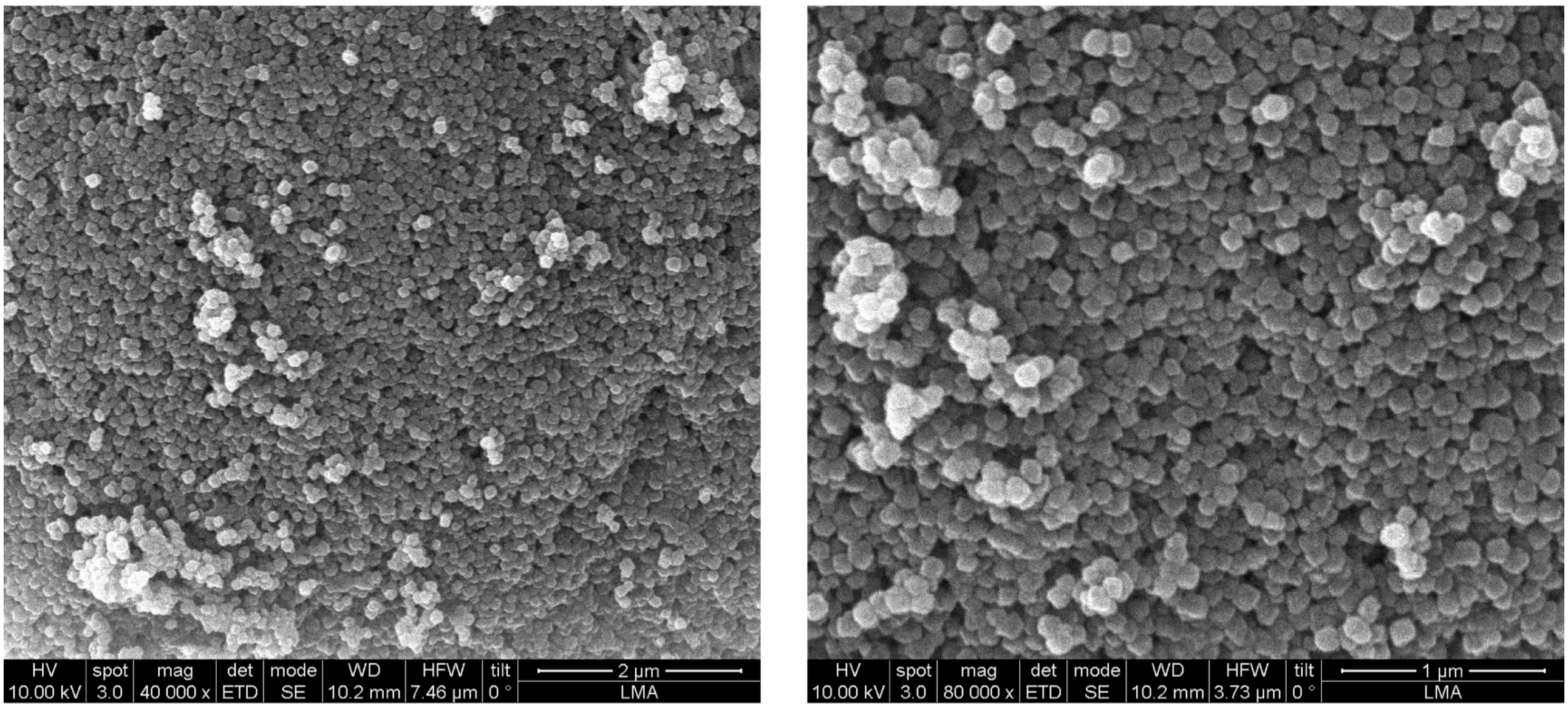

(VO)$_{0.04}$dPCN-224

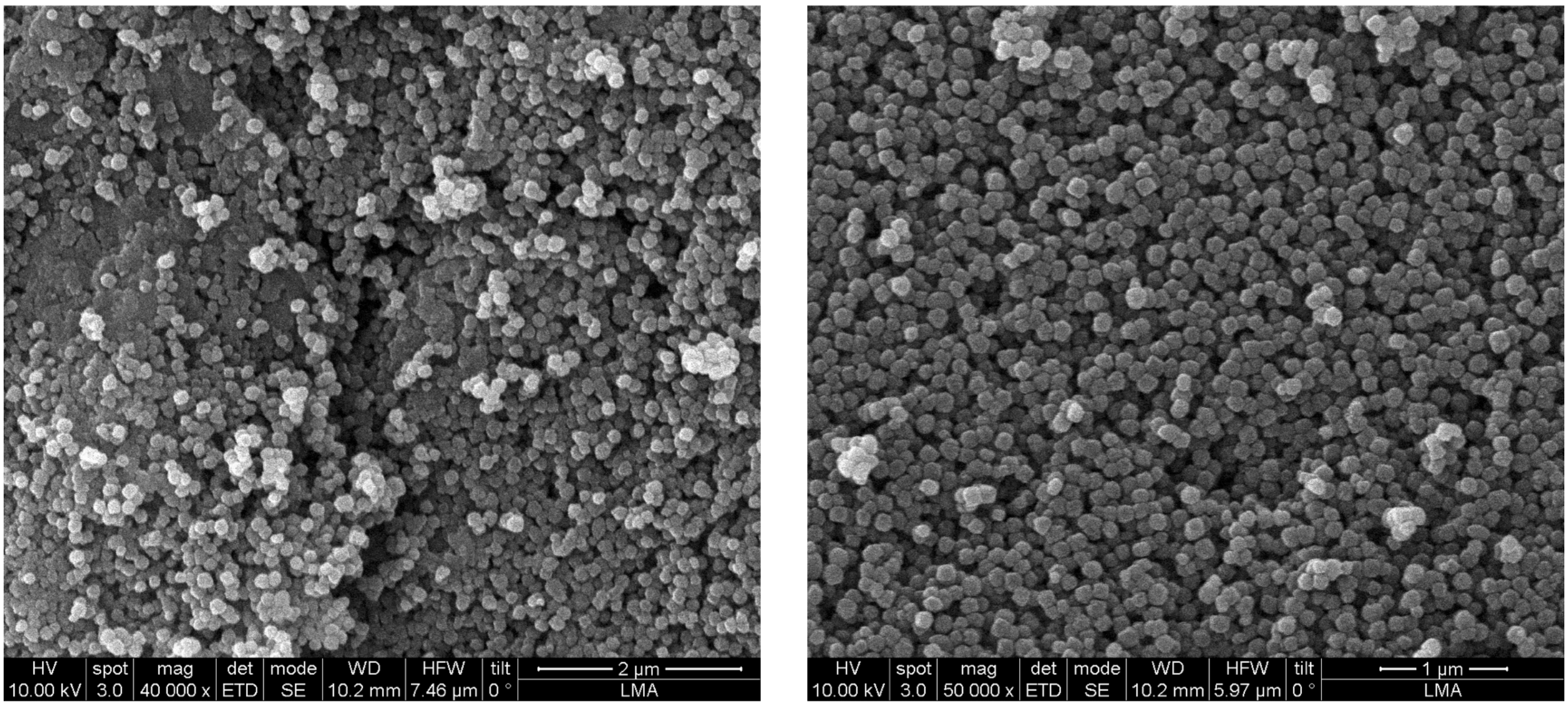

Figure S6. Additional SEM images of (M)$_x$dPCN-224 NPs obtained along the conditions of entries 8 and 9 in Table S1.

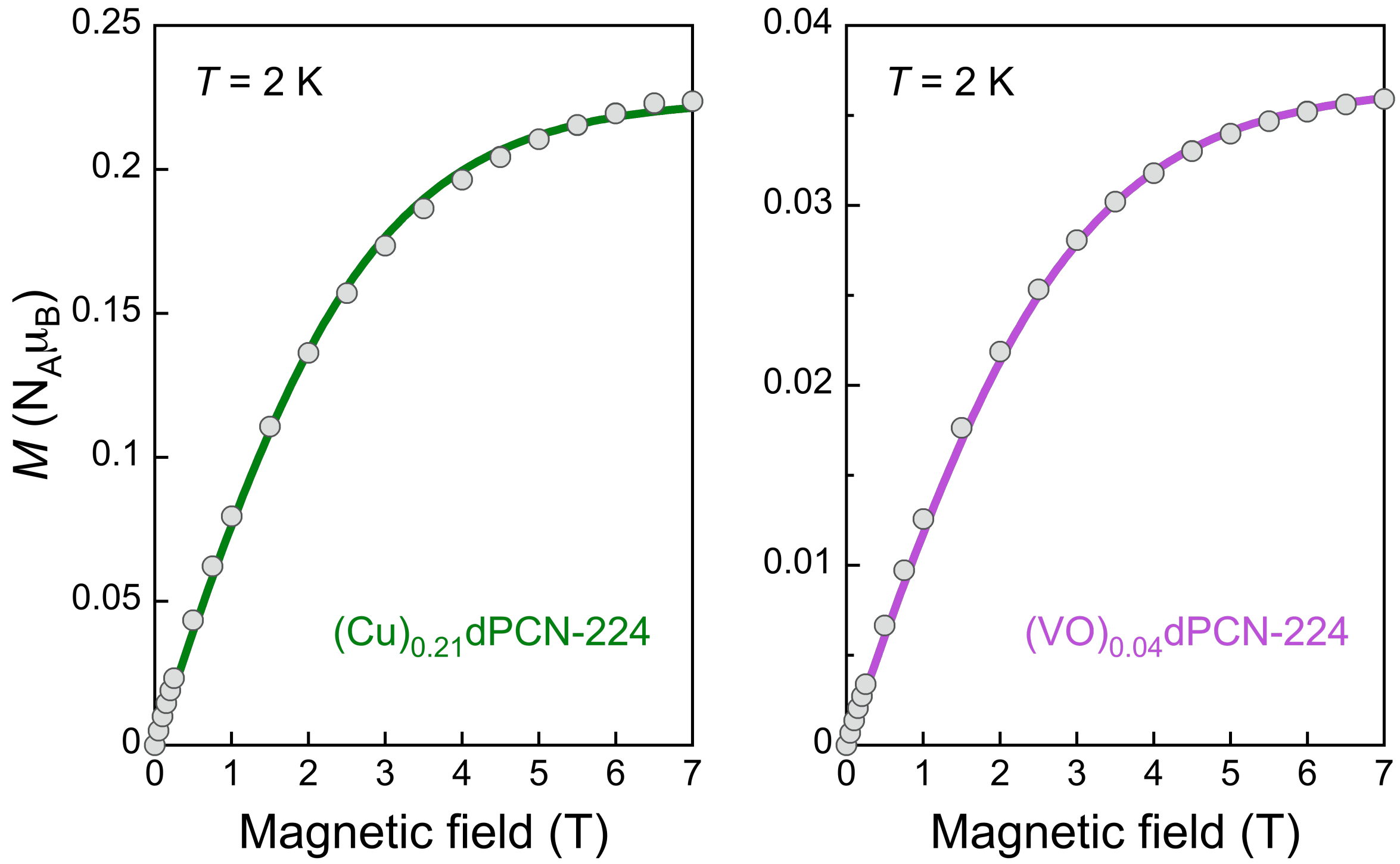

Figure S7. Magnetization isotherms at 2 K for $(Cu)_{0.21}$dPCN-224 (left, entry 8 in Table S1) and $(VO)_{0.04}$dPCN-224 (right, entry 9 in Table S1) nanoparticles. The full green and violet lines are the Brillouin function for T = 2 K, S = 1/2 and g_{eff} = 2.108 / 1.981 scaled by a factor 0.213 / 0.037, respectively. Note that the formula weight used to calculate the molar magnetizations takes into account the indicated Cu / VO content.

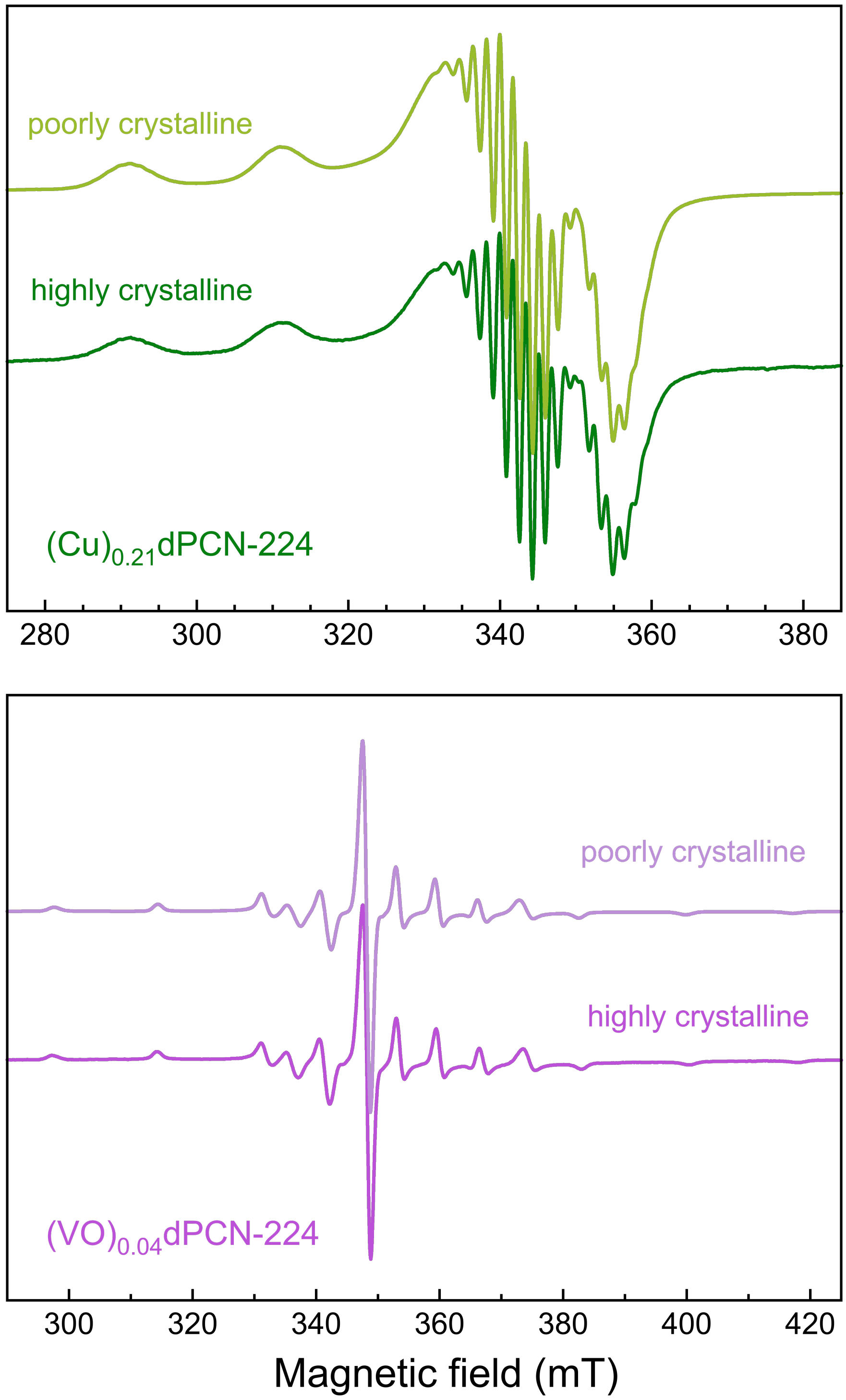

Figure S8. Comparison of cw-EPR spectra for highly crystalline and poorly crystalline samples of Cu (top) and VO (bottom) doped dPCN-224 NPs.

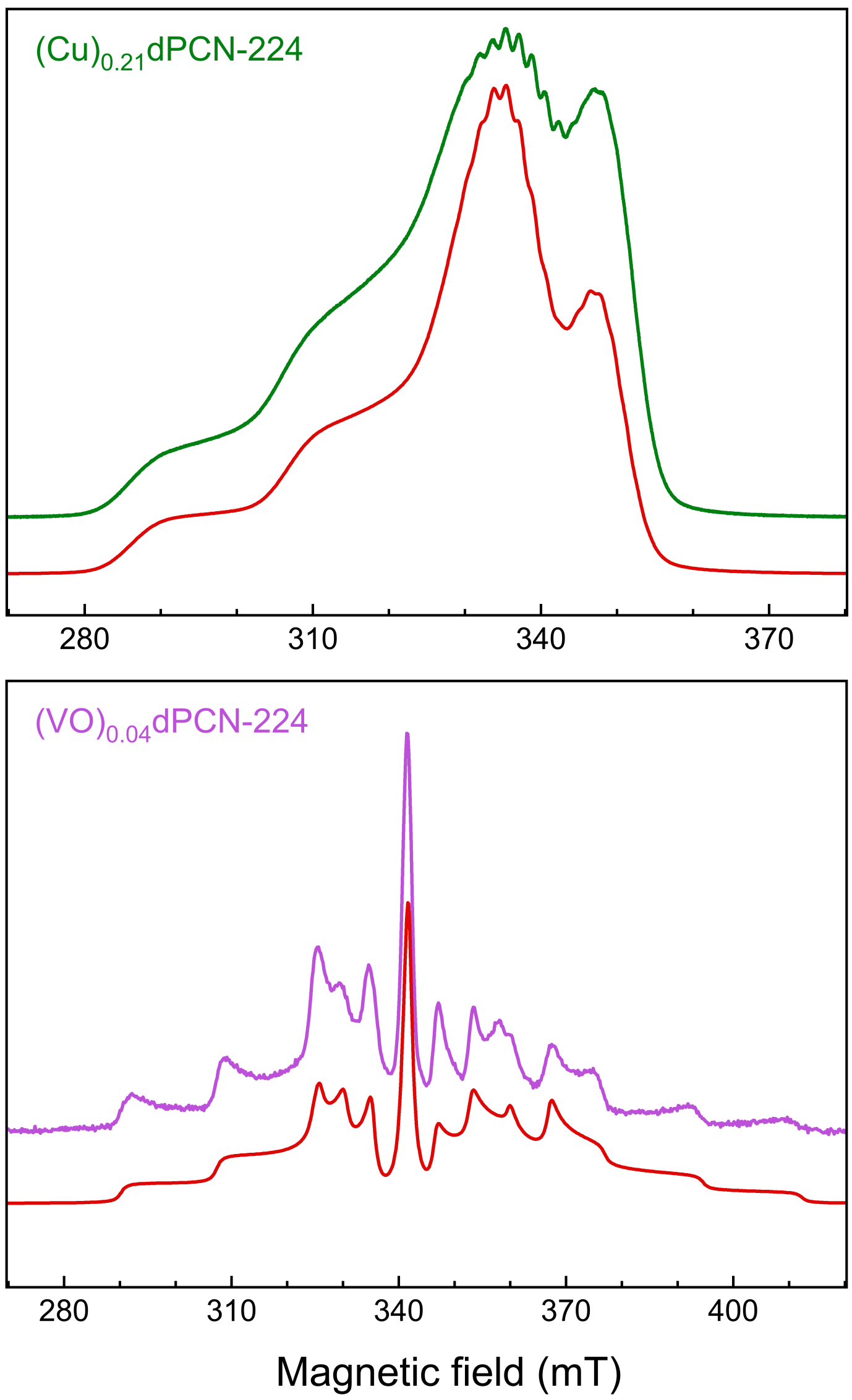

Figure S9. 2p-ESE-detected field-swept (EDFS) spectra at 4 K of $(Cu)_{0.21}$dPCN-224 (top, green line, 9.706024 GHz) and $(VO)_{0.04}$dPCN-224 (bottom, purple line, 9.672684 GHz), together with simulations done using the same SH parameters as for the cw spectra.

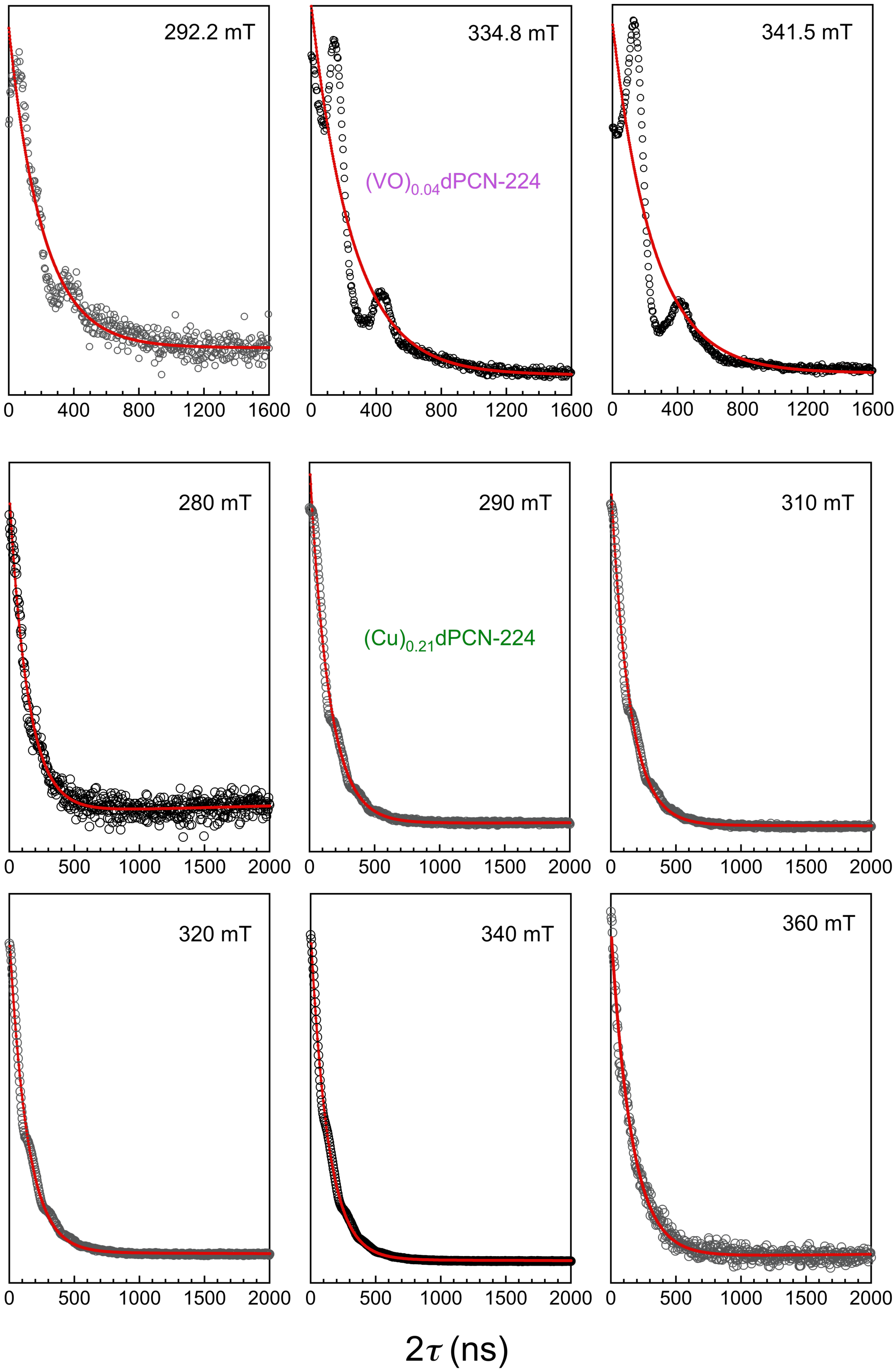

Figure S10. 2p ESE intensity as a function of inter-pulse interval, τ, for $(Cu)_{0.21}$dPCN-224 (green-grey circles) and $(VO)_{0.04}$dPCN-224 at the indicated applied magnetic fields and T = 4 K. Grey lines correspond to the evolution predicted with an exponential decay expressed as:

$$y(\tau) = y_0 + A_{2p}e^{-2\tau/T_M}$$

with the values of T_M that give the best fit gathered in Table S2.

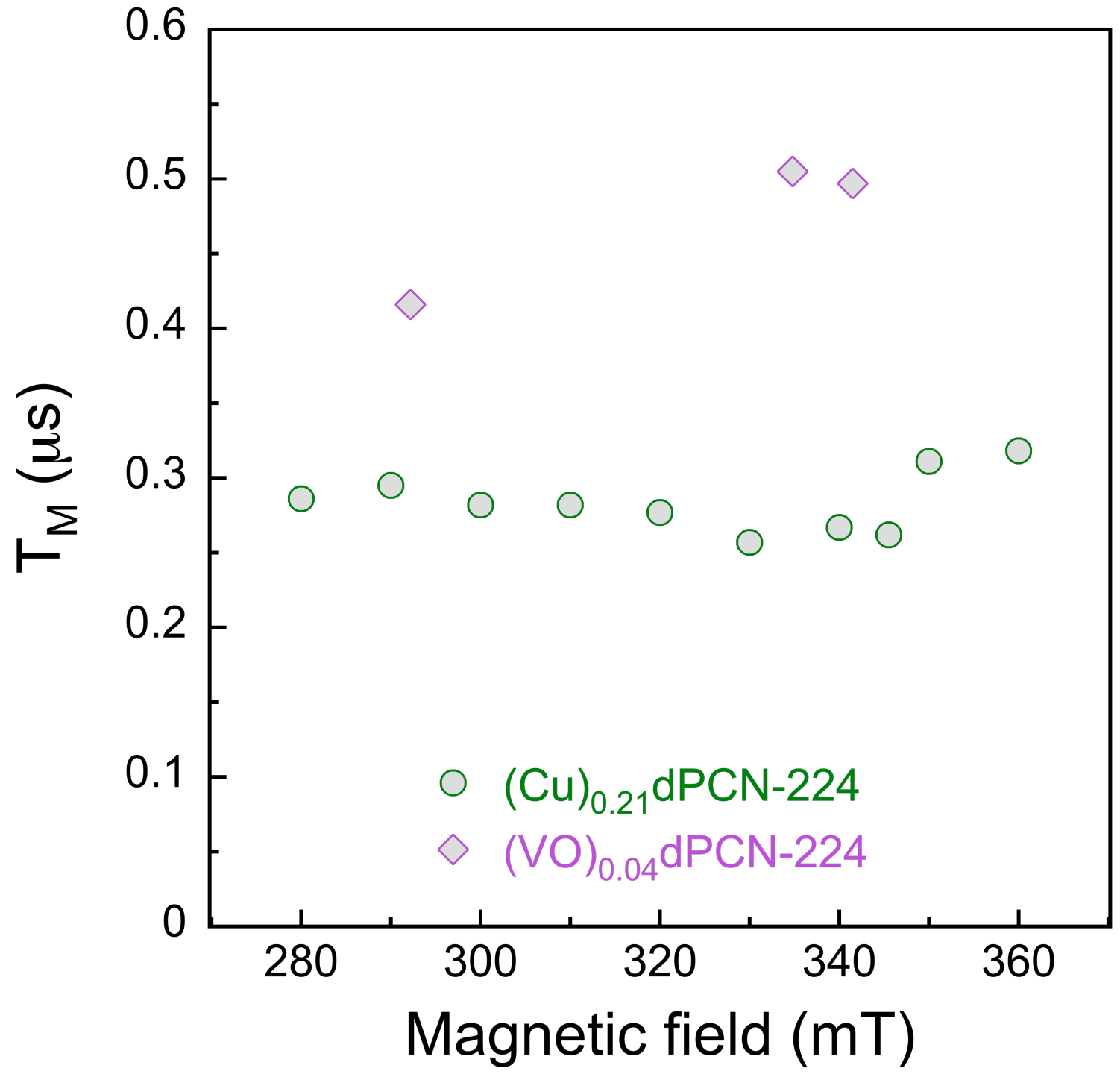

Figure S11. Phase memory times T_M of $(Cu)_{0.21}$dPCN-224 (green-grey circles) and $(VO)_{0.04}$dPCN-224 (purple-grey rhombs) NPs derived from Hahn-echo decays at various applied magnetic fields.

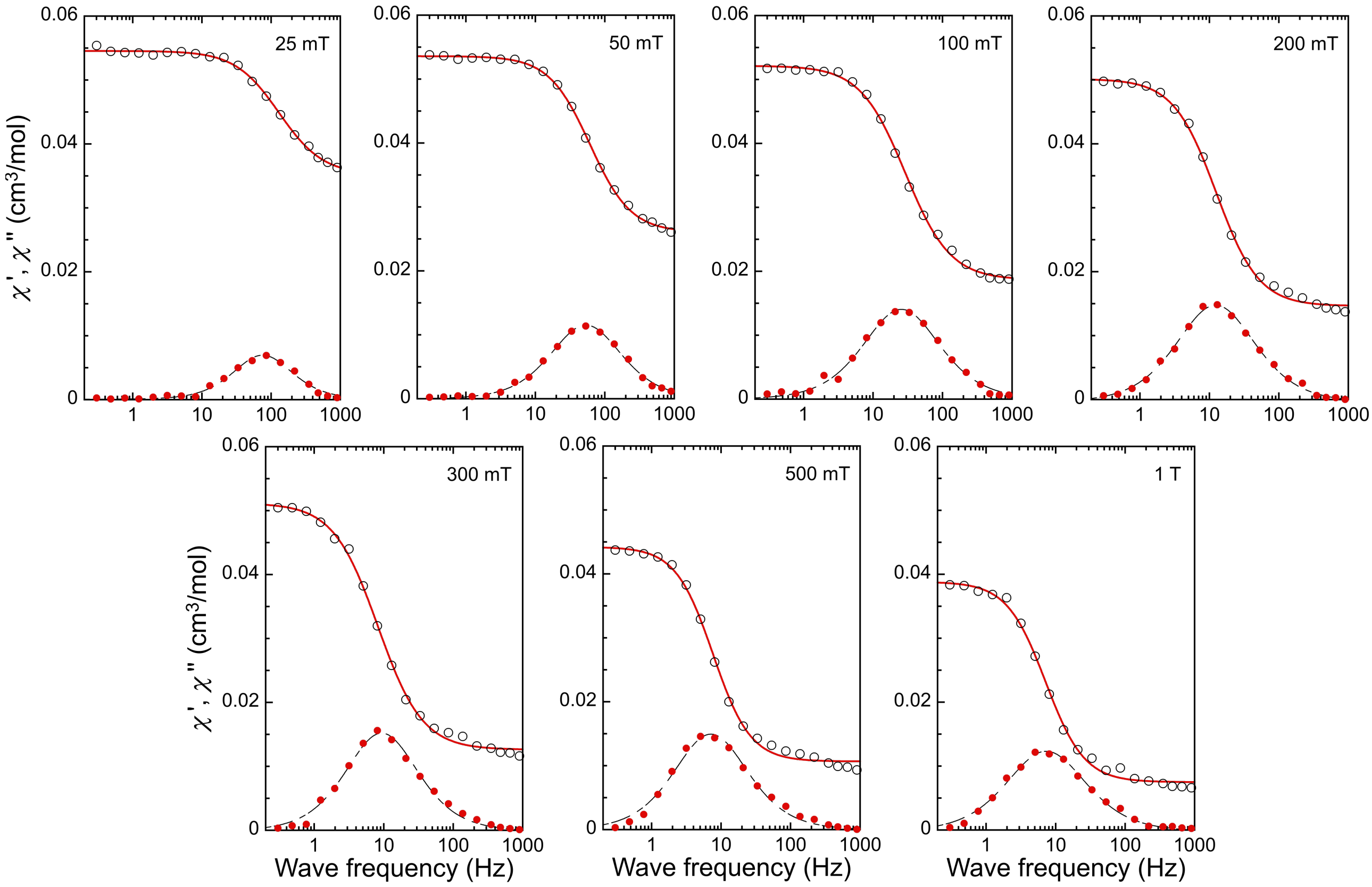

Figure S12. Frequency dependence of the in-phase (empty black symbols) and out-of-phase (full red symbols) *ac* magnetic susceptibility of $(Cu)_{0.21}$dPCN-224 NPs at 2 K and increasing applied *dc* fields as indicated. Lines are fits of the generalized Debye model expressions of the real and imaginary components of the *ac* susceptibility, χ' and χ'' to the experimental data, allowing to derive the characteristic relaxation times τ (Table S2):

$$\chi'(\omega) = \chi_S + (\chi_T - \chi_S)\frac{1 + (\omega\tau)^\beta \cos\left(\frac{\pi\beta}{2}\right)}{1 + 2(\omega\tau)^\beta \cos\left(\frac{\pi\beta}{2}\right) + (\omega\tau)^{2\beta}}$$

$$\chi''(\omega) = (\chi_T - \chi_S)\frac{(\omega\tau)^\beta \sin\left(\frac{\pi\beta}{2}\right)}{1 + 2(\omega\tau)^\beta \cos\left(\frac{\pi\beta}{2}\right) + (\omega\tau)^{2\beta}}$$

where ω is the angular frequency, χ_T the isothermal susceptibility, χ_S the adiabatic susceptibility and β describes the distribution of relaxation times.

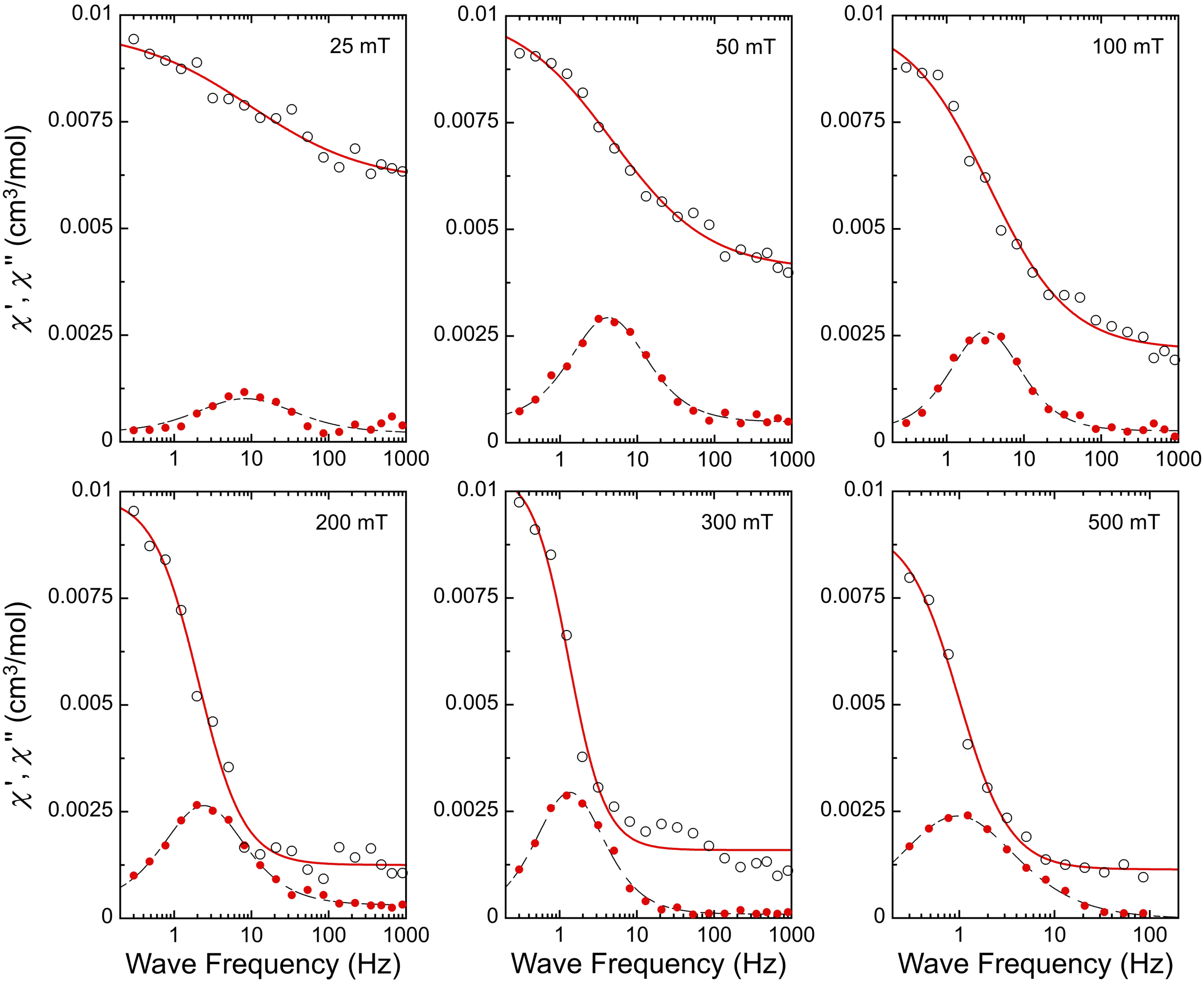

Figure S13. Frequency dependence of the in-phase (empty black symbols) and out-of-phase (full red symbols) *ac* magnetic susceptibility of $(VO)_{0.04}$dPCN-224 NPs at 2 K and increasing applied *dc* fields as indicated. Lines are fits of the generalized Debye model expressions of the real and imaginary components of the *ac* susceptibility (see caption of Figure S12), enabling to derive the characteristic relaxation times τ (Table S2).

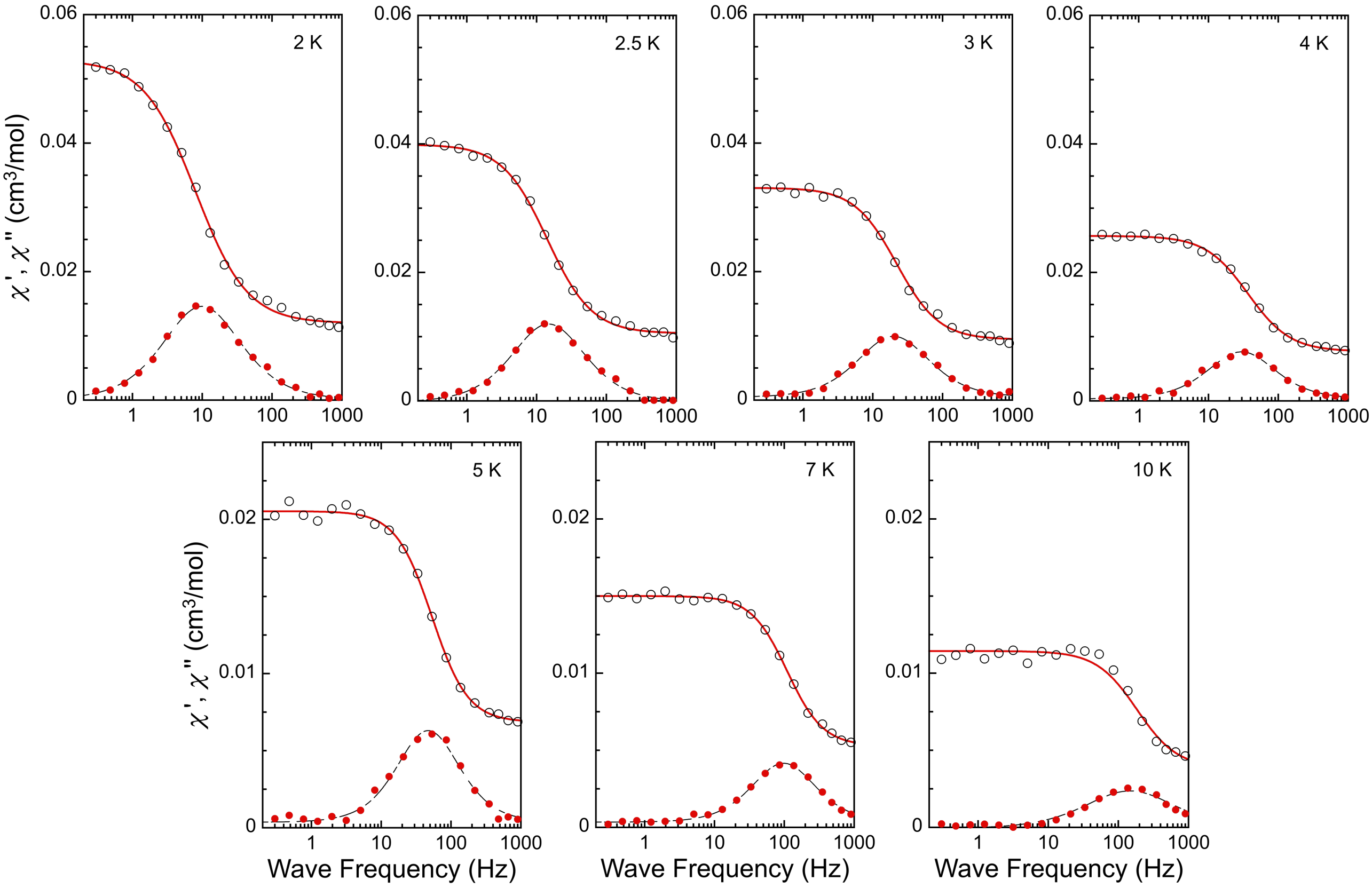

Figure S14. Frequency dependence of the in-phase (empty black symbols) and out-of-phase (full red symbols) *ac* magnetic susceptibility of $(Cu)_{0.21}$dPCN-224 NPs at 300 mT and increasing temperature as indicated. Lines are fits of the generalized Debye model expressions of the real and imaginary components of the *ac* susceptibility (see caption of Figure S12), enabling to derive the characteristic relaxation times τ (Table S2).

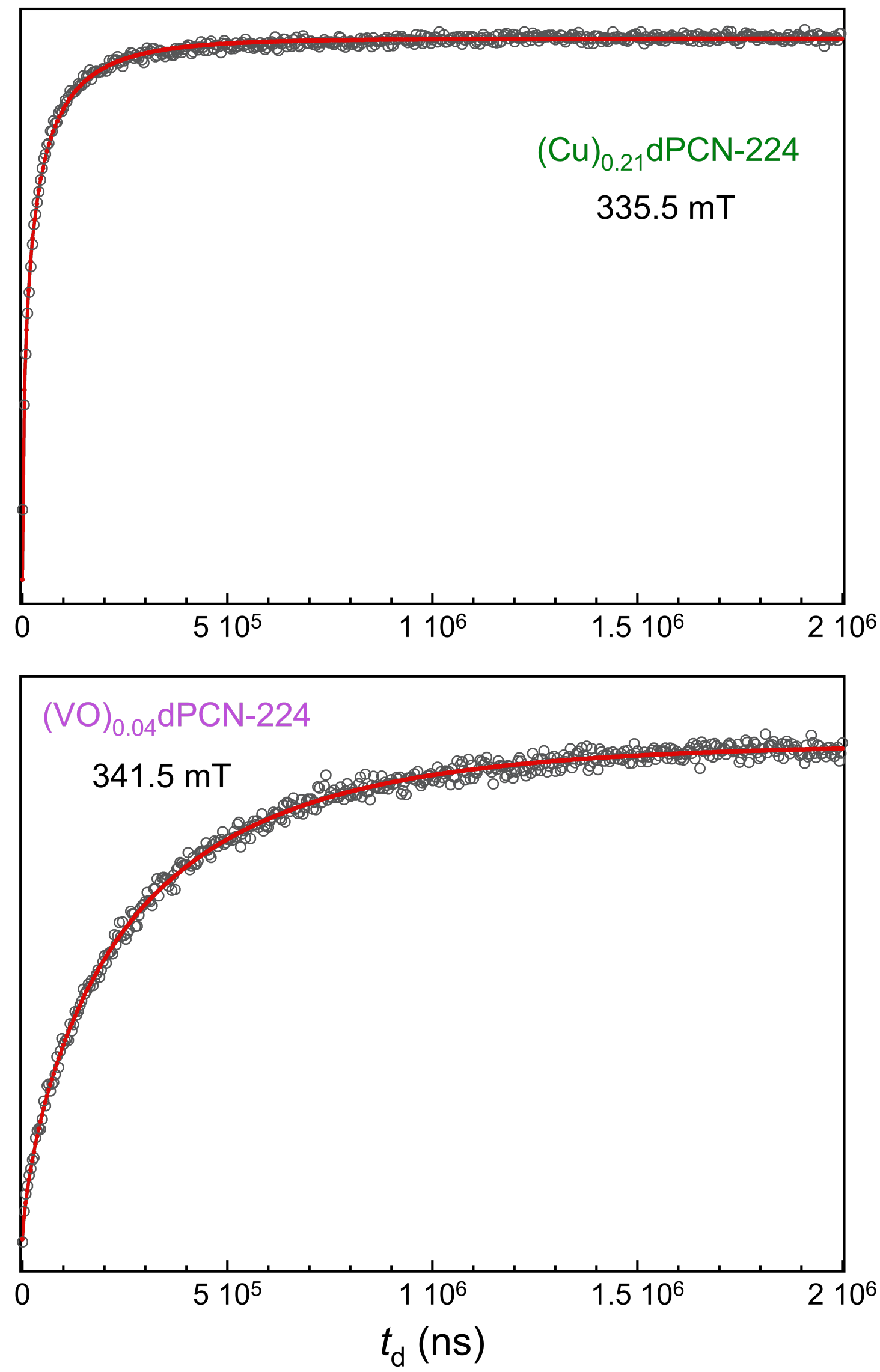

Figure S15. Normalized ESE detected Inversion Recovery as a function of delay time, t_d, for $(Cu)_{0.21}$-dPCN-224 (top) and $(VO)_{0.04}$-dPCN-224 (bottom) NPs at the indicated applied magnetic fields (circles). Red lines correspond to a stretched exponential t_d dependence modeled by:

$$y(t_d) = y_\infty - y_0 e^{-\left(t_d/\beta T_1'\right)^\beta}$$

that best fit to the data (Table S2).

Table S3. Parameters describing the relaxation processes in $(Cu)_{0.21}$dPCN-224 and $(VO)_{0.04}$dPCN-224 NPs, as derived from 2p Hahn echo decays (Figure S10), frequency dependence of the ac susceptibility (Figures S11-S14) and Inversion Recovery experiments (Figure S15).

	Magnetic field (mT)	*T* (K)	T_M (μs)	τ (≡T_1, ms)	T_1^{fast} (≡T_1', ms)	β
$(Cu)_{0.21}$dPCN-224	280	4	0.286(4)			
	290	4	0.295(2)			
	300	4	0.282(2)			
	310	4	0.282(2)			
	320	4	0.277(2)			
	330	4	0.257(1)			
	340	4	0.267(1)			
	345.5	4	0.262(1)			
	350	4	0.311(1)			
	360	4	0.318(3)			
	25	2		1.66(3)		
	50	2		2.79(2)		
	100	2		5.86(1)		
	200	2		12.83(1)		
	300	2		18.19(1)		
	500	2		21.59(1)		
	1000	2		22.01(2)		
	300	2		18.01(1)		
	300	2.5		11.03(1)		
	300	3		7.63(1)		
	300	4		4.82(2)		
	300	5		3.18(2)		
	300	7		1.53(3)		
	300	10		0.98(5)		
	335.5	4			0.049(1)	0.52(1)
$(VO)_{0.04}$dPCN-224	292.2	4	0.416(10)			
	334.8	4	0.505(16)			
	341.5	4	0.497(20)			
	25	2		17.78(5)		
	50	2		35.72(3)		
	100	2		47.74(3)		
	200	2		71.55(3)		
	300	2		118.22(4)		
	500	2		163.36(6)		
	1000	2		203.0(1)		
	341.5	4			0.344(2)	0.74(1)

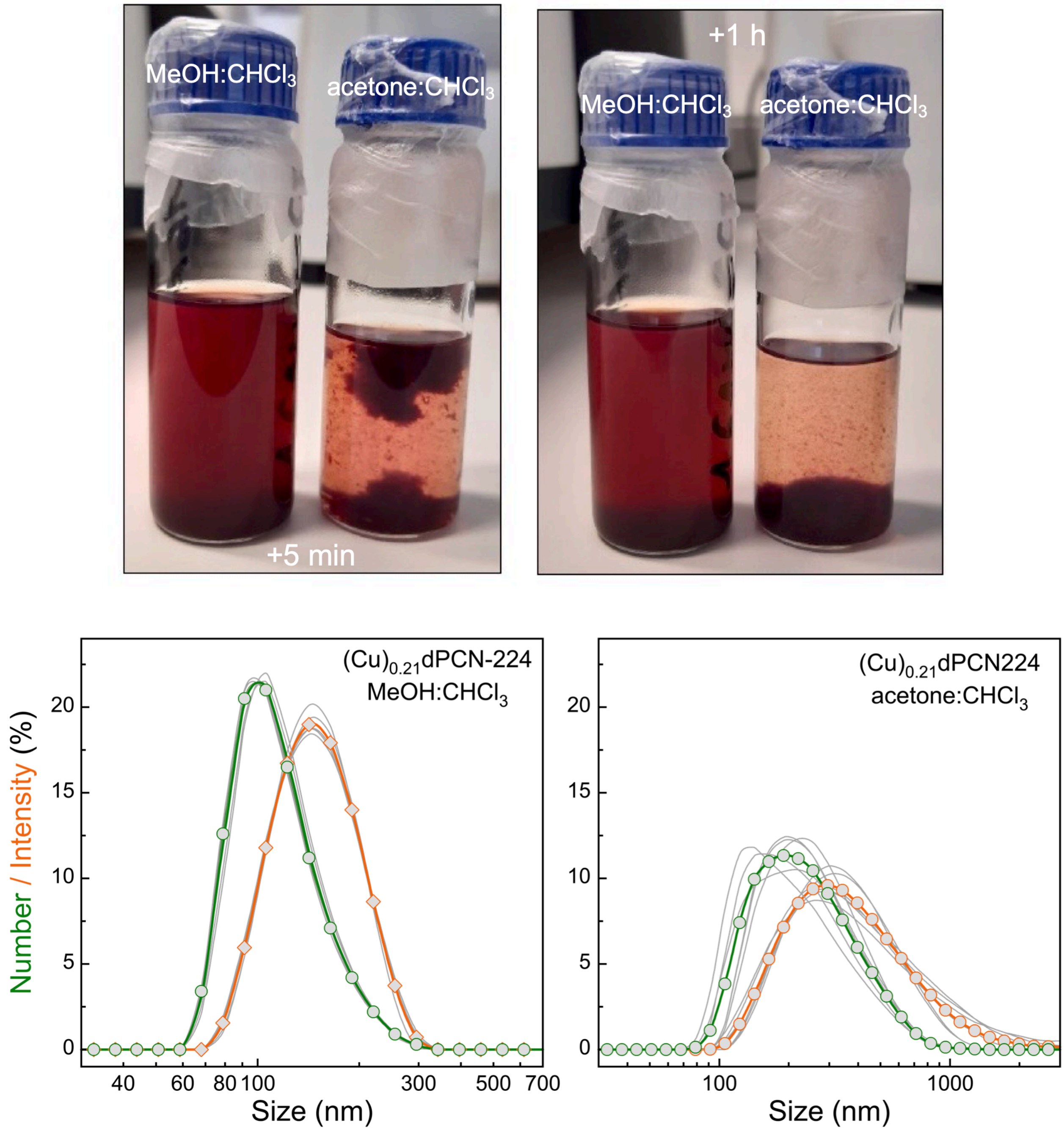

Figure S16. Top: Appearance of the 1:3 MeOH:$CHCl_3$ (left vial) and acetone:$CHCl_3$ (right vial) dispersions of $(Cu)_{0.21}$dPCN-224 NPs 5 and 60 min after dilution with $CHCl_3$ from the respective MeOH and acetone dispersions. Bottom: Dynamic Light Scattering measurements on the same dispersions. Grey lines are two groups of 3 consecutive measurements, also done about 5 and 60 min after dilution with $CHCl_3$, while thicker colored lines are the average of all 6 measurements.

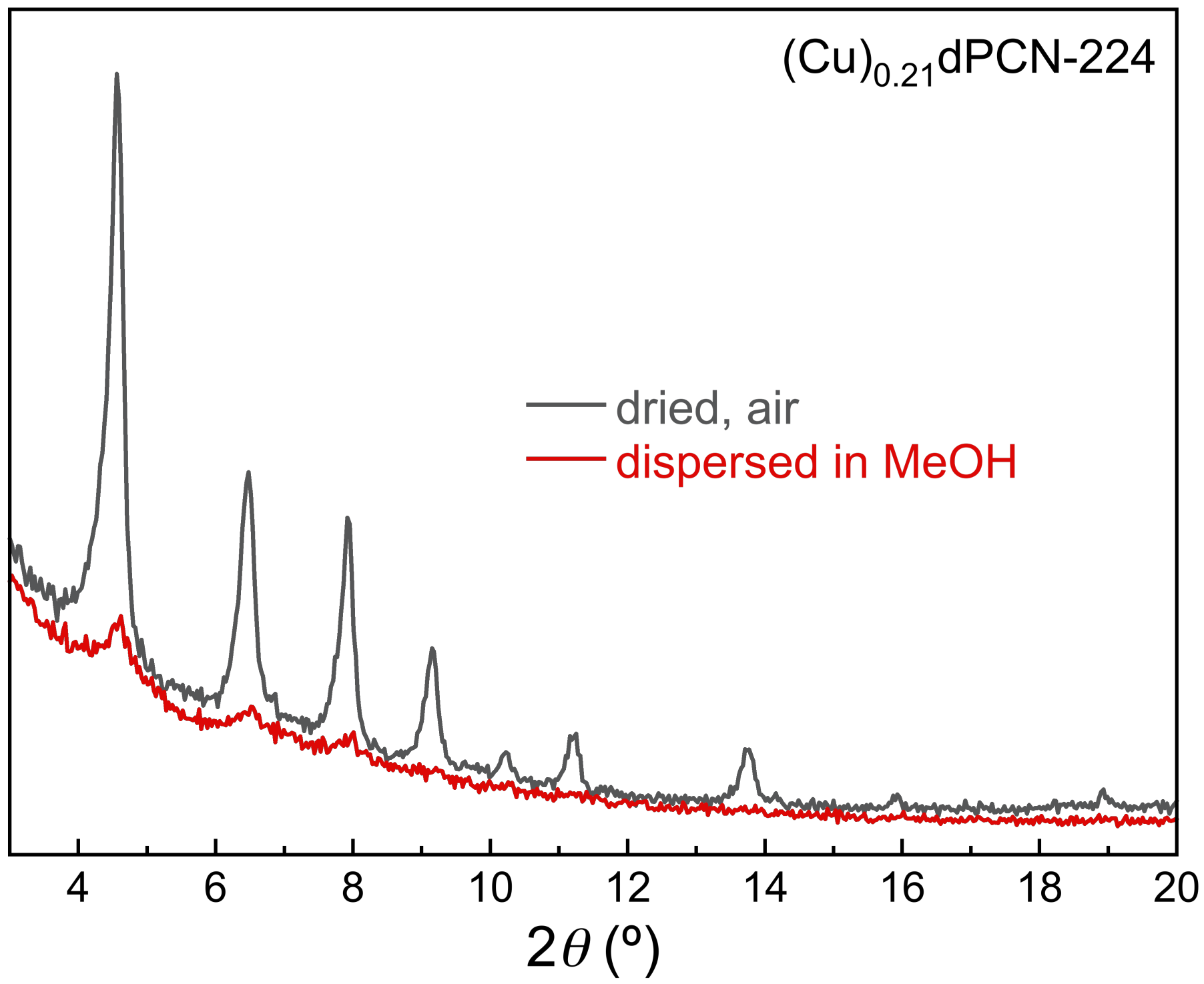

Figure S17. Powder X-Ray Diffractograms of two fractions of the same $(Cu)_{0.21}$dPCN-224 NPs : the grey lines corresponds to NPs dried from the acetone dispersions and kept in air for > 4 weeks, while the red line corresponds to NPs redispersed in MeOH and kept in that dispersion for > 3 weeks, before being recovered by evaporation of the solvent, and dried in air.

Langmuir and Langmuir−Blodgett film fabrication

Surface pressure−area (π−A) isotherms were obtained using a Teflon Langmuir trough NIMA model 702 (dimensions 720 mm x 100 mm).

Brewster Angle Microscopy (BAM) images were acquired using a KSV NIMA Micro BAM equipped with a red laser (659 nm, 50 mW) as the light source. The incidence angle was fixed at 53.1°, and a black quartz plate was placed inside the trough as a light trap. The optics of the system provided a spatial resolution of 6 μm per pixel in the water surface plane and a field of view of 3600x4000 μm^2.

Langmuir-Blodgett and Langmuir-Schaefer films were made with a KSV-NIMA trough model KN 2003, with dimensions of 580 mm × 145 mm.

In all cases, compression was performed by a symmetric double-barrier system at constant speed of 8 $cm^2 \cdot min^{-1}$. Surface pressure was continuously monitored during the experiments by means of a Wilhelmy balance using a filter paper plate. The troughs are housed in a clean room inside closed cabinets and the room temperature is maintained at 293 K (±1 K). Ultra-pure Milli-Q water (ρ = 18.2 MΩ·cm) was employed as subphase. In all experiments, the trough was carefully cleaned with acetone and chloroform, filled and emptied twice with Milli-Q water and ultimately filled with Milli-Q water. The subphase was then carefully cleaned by closing the barriers down to 40 mm distance and mild surface-touch vacuuming intra-barriers area. After opening the barriers to the maximum area, the system was let to equilibrium for 5 minutes. The dPCN224 or M-dPCN224 NPs dispersion was carefully spread drop-by-drop onto the subphase using a Hamilton microsyringe held very close to the subphase surface, and left to evaporate for 20 minutes before starting the compression. Substrates (Si wafer) were cleaned by ultrasonication, successively in chloroform, acetone and ethanol. Transfers were carried out by vertical-dipping at various surface pressures, the substrate being approached to the surface at 0.2 $mm \cdot min^{-1}$ and raised at 10 $mm \cdot min^{-1}$.

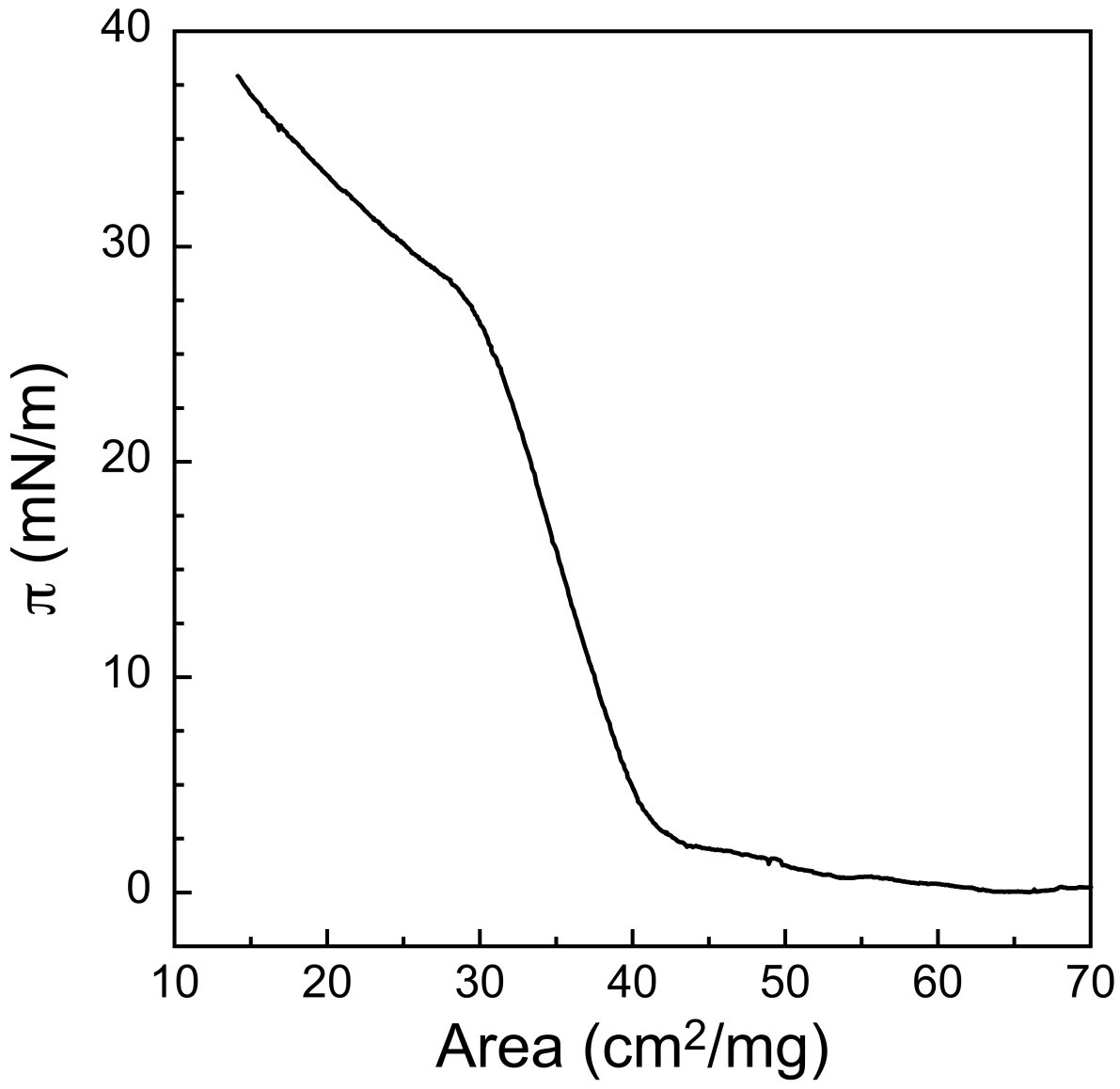

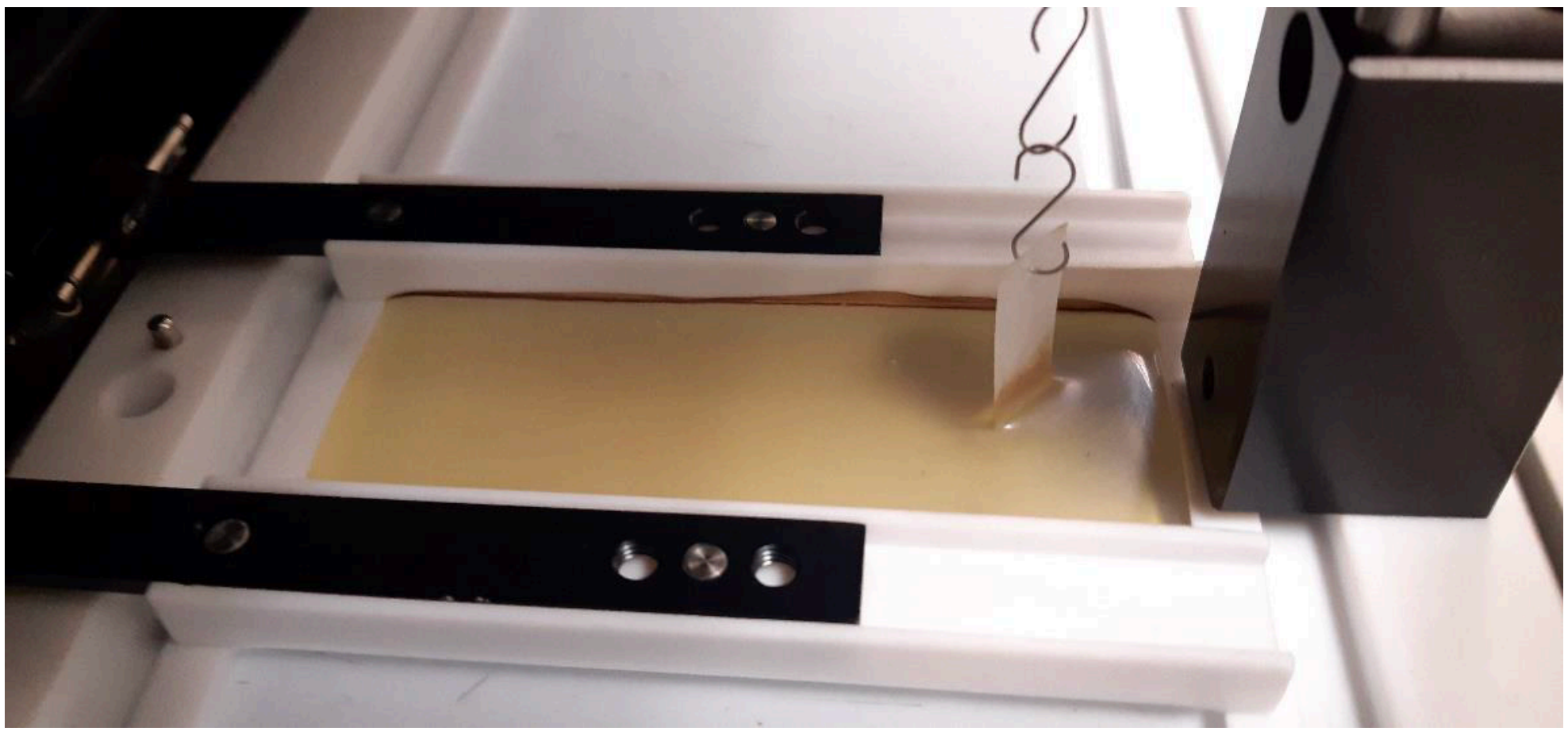

Figure S18. Top: Representative surface-pressure isotherm obtained when using not-ideal dPCN-224 dispersions. The change of slope indicates the instability of the Langmuir film formed upon compression, while the slope before is also less steep than when using good dispersions. Bottom: picture of the Langmuir trough at low areas showing the accumulation of material on the barriers due to the instability of the film formed from not-ideal dispersions.

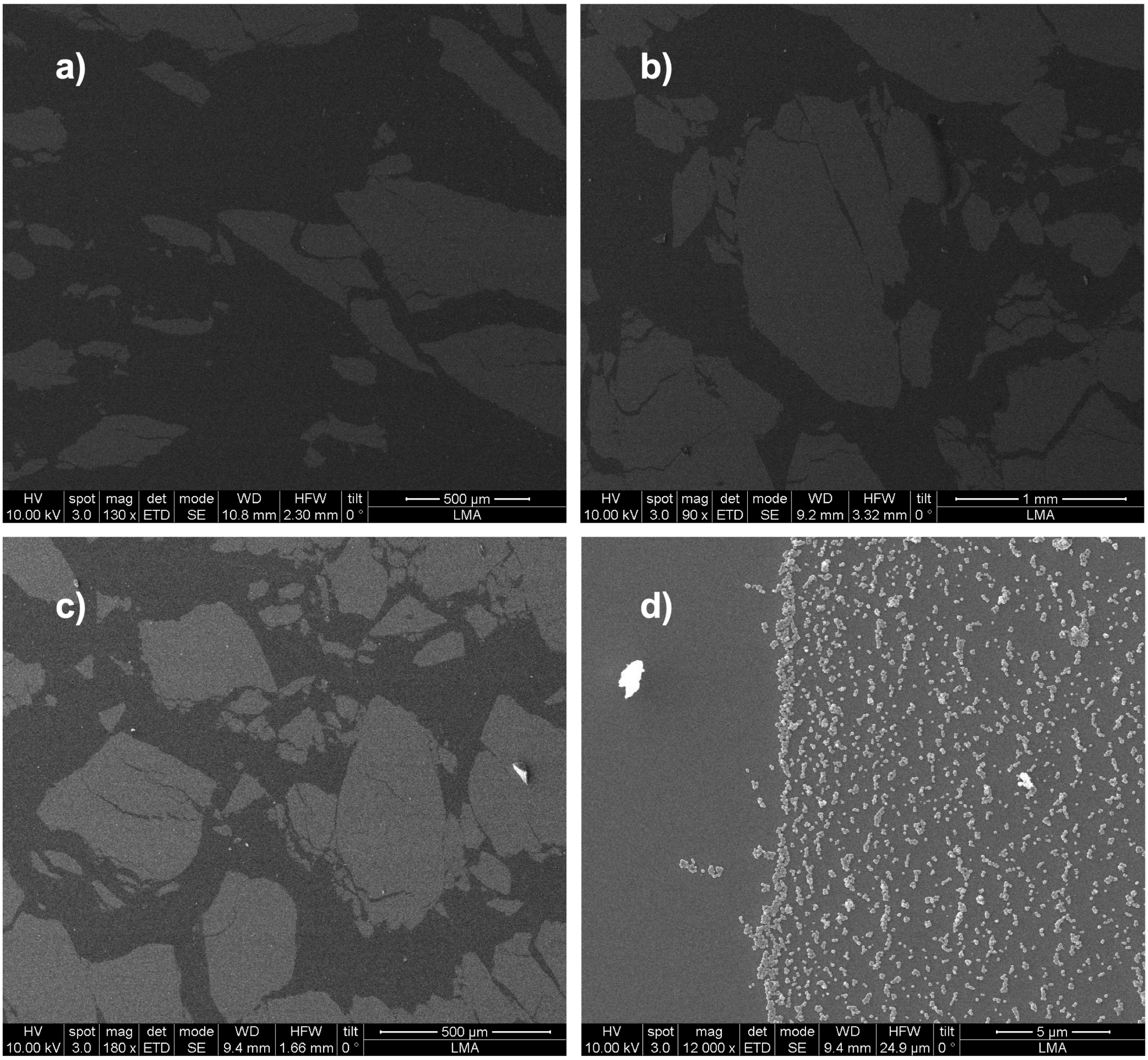

Figure S19. Representative SEM images of Langmuir-Blodgett transfers on Si, showing incomplete coverage at 20, 30, 36 mN/m (a-c), indicating the Langmuir films breaks upon LB transfer. The image in d) shows a detail of the part of the substrate that first leaves the subphase.

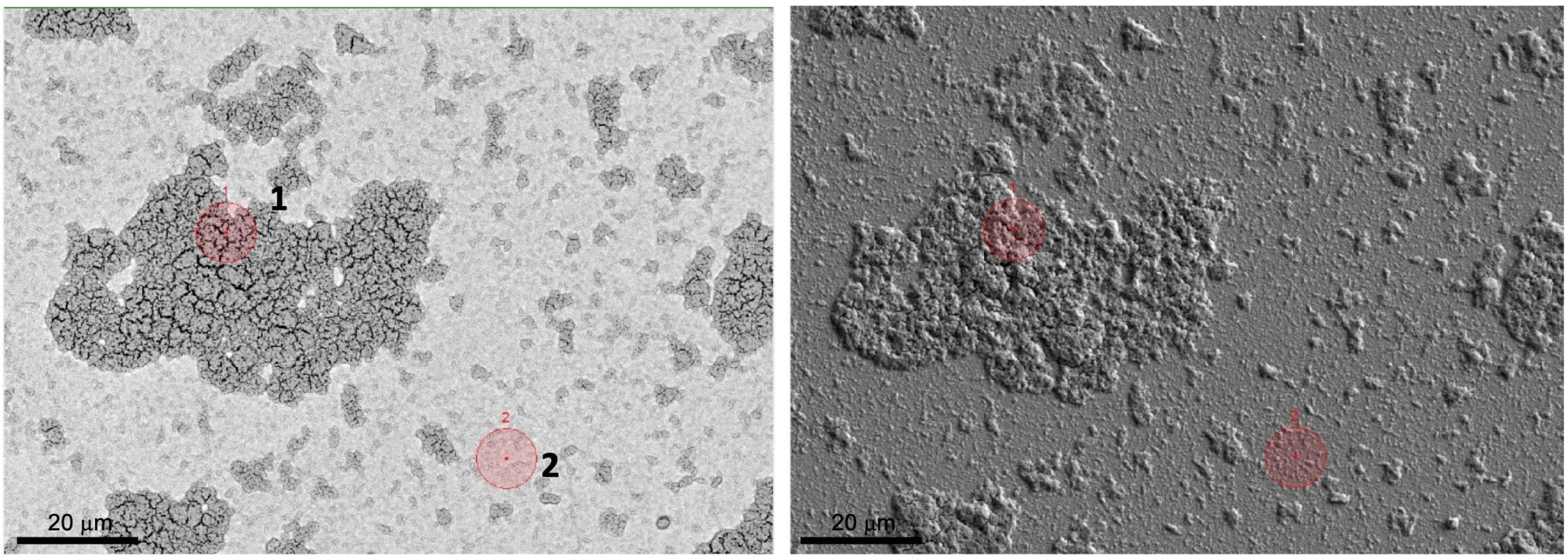

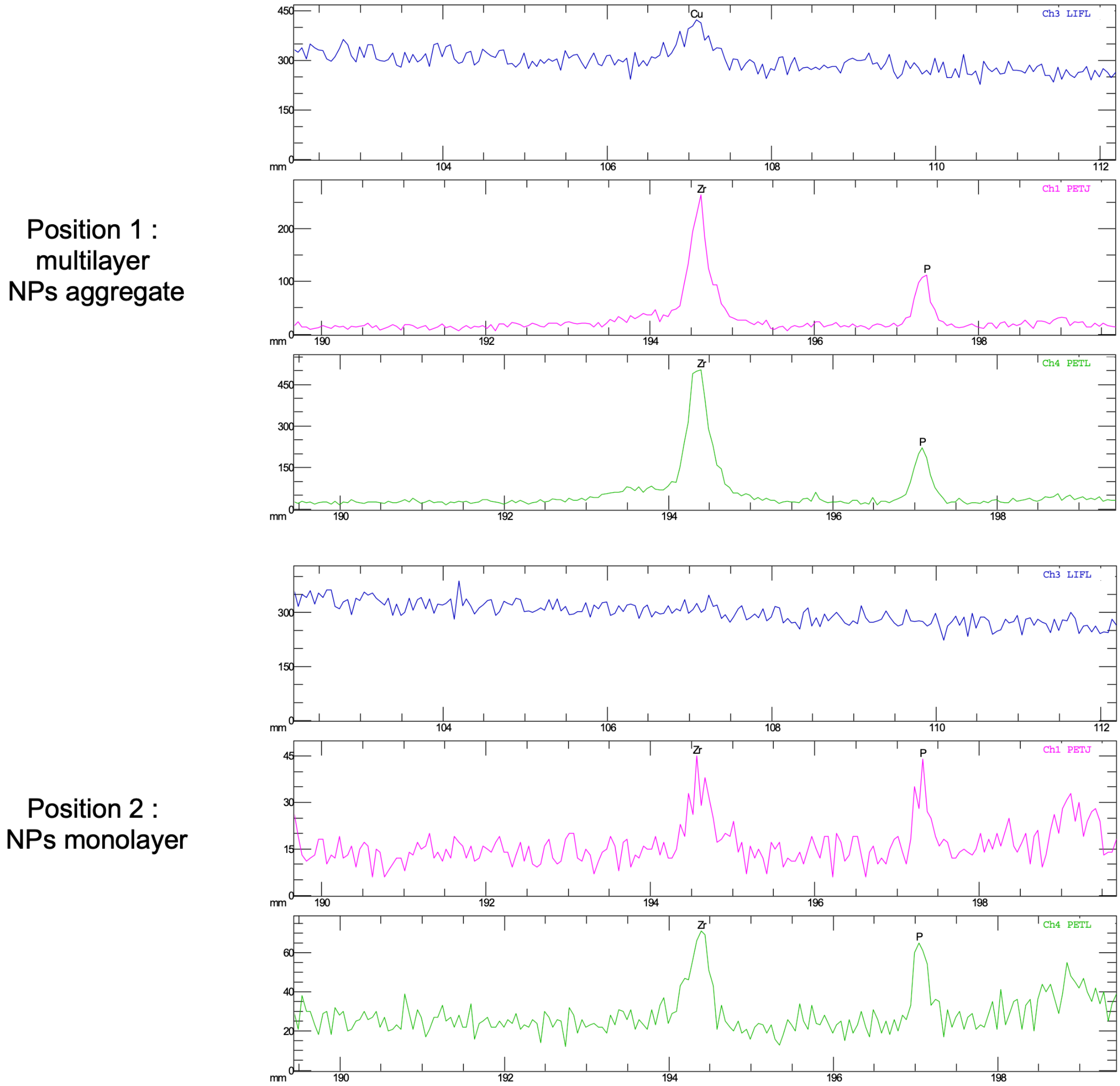

Figure S20. Surface microanalysis of $(Cu)_{0.21}$dPCN-224 NPs film on Si. Top: SEM images indicating the two positions analyzed, a multilayer NPs aggregate (1) and an area with only NPs monolayer (2). Bottom: LIFL, PETJ and PETL channels at the two positions analyzed, showing the detection of Zr and Cu. In monolayer areas, Cu is hardly detected, due to the diluted nature of the NPs and the set-up sensitivity.

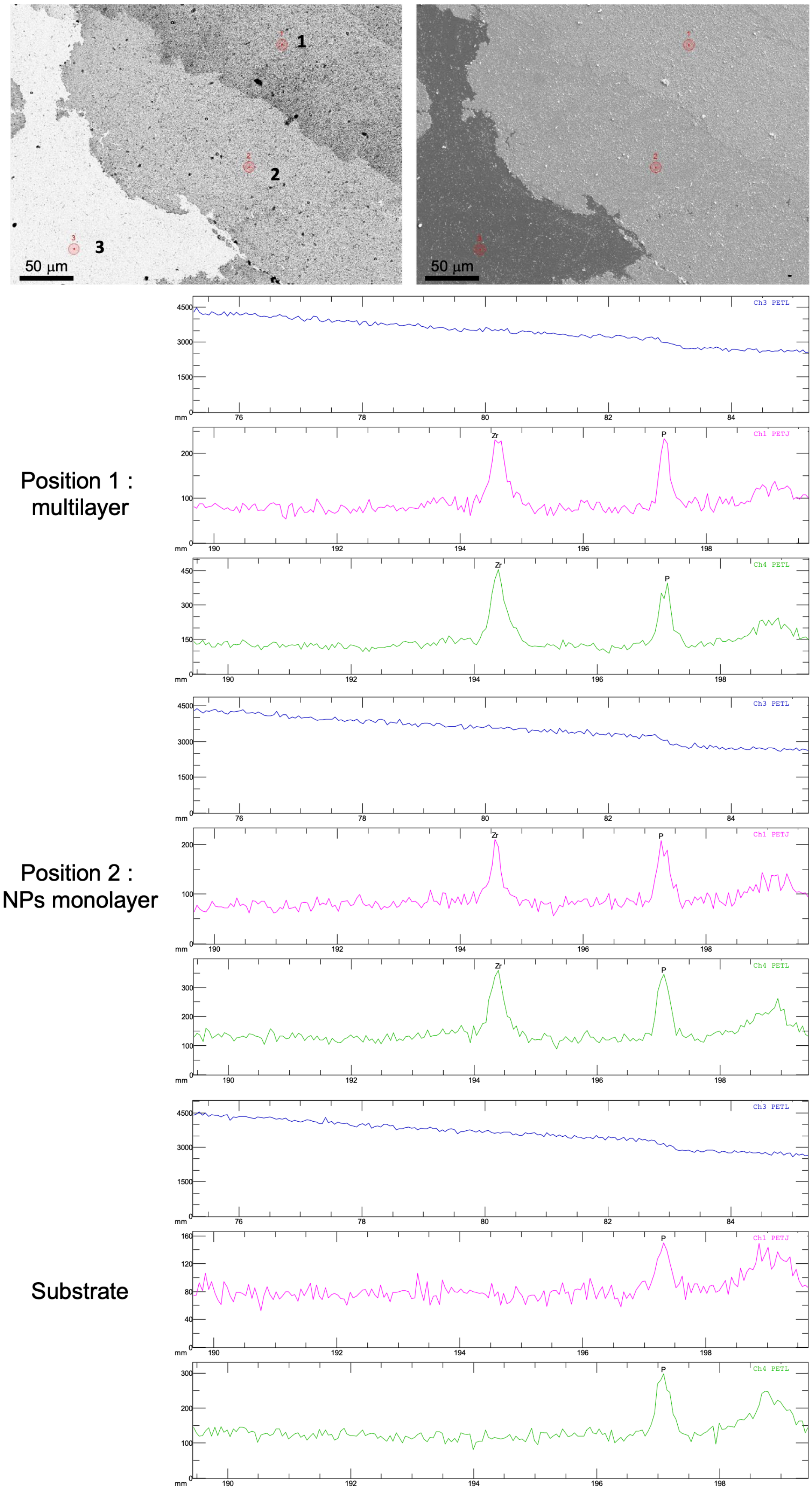

Figure S21. Surface microanalysis of $(VO)_{0.04}$dPCN-224 NPs film on Si. Top: SEM images indicating the three positions analyzed, a multilayer (1) an area with NPs monolayer (2) and the substrate (3). Bottom: LIFL, PETJ and PETL channels at the three positions analyzed, showing the detection of Zr. V is not detected, due to the too diluted nature of the NPs and the set-up sensitivity.

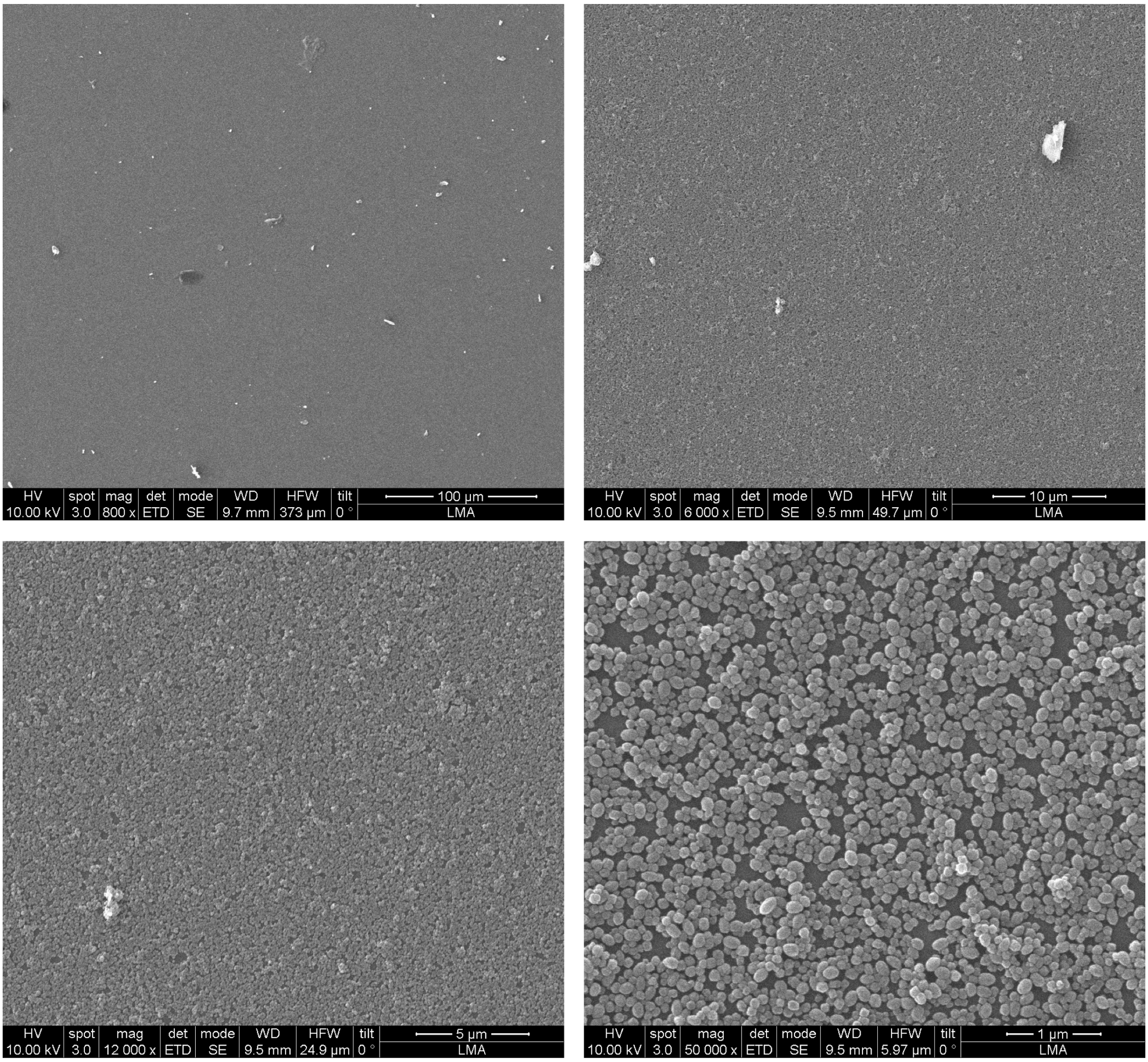

Figure S22. Representative SEM images of $(M)_x$dPCN-224 films transferred on Si through reverse Langmuir Schaefer conditions at 25 mN/m (here M=VO and x=0.04).

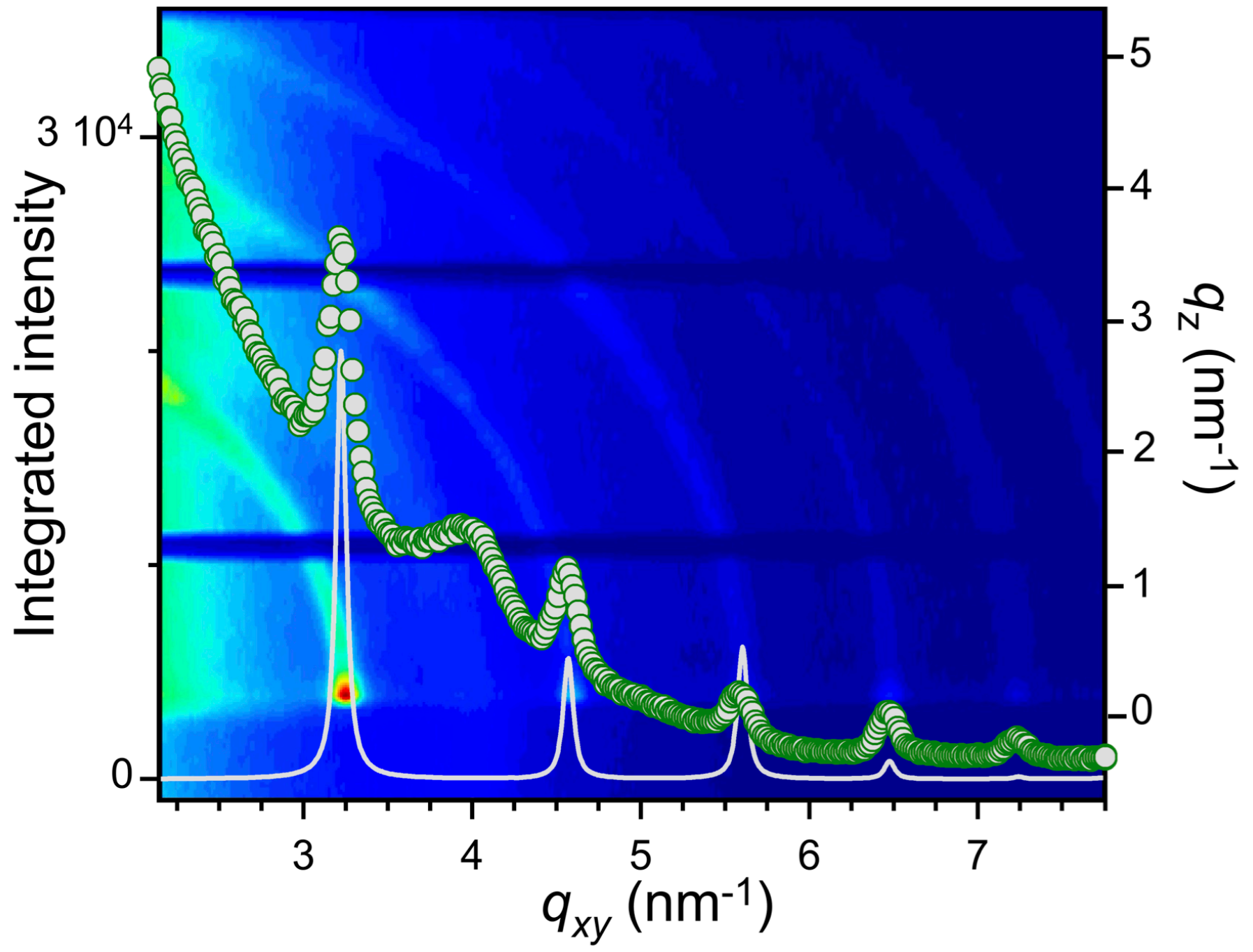

Figure S23. GIXD patterns of a $(Cu)_{0.21}$dPCN-224 NPs film transferred on Si by inverse-Langmuir-Schaefer (green-grey symbols). The white line are the patterns calculated from the reported single-crystal structure of dPCN224 (grey). These *I*(q) data are derived from the full 2D plot shown as background, by integrating radially.

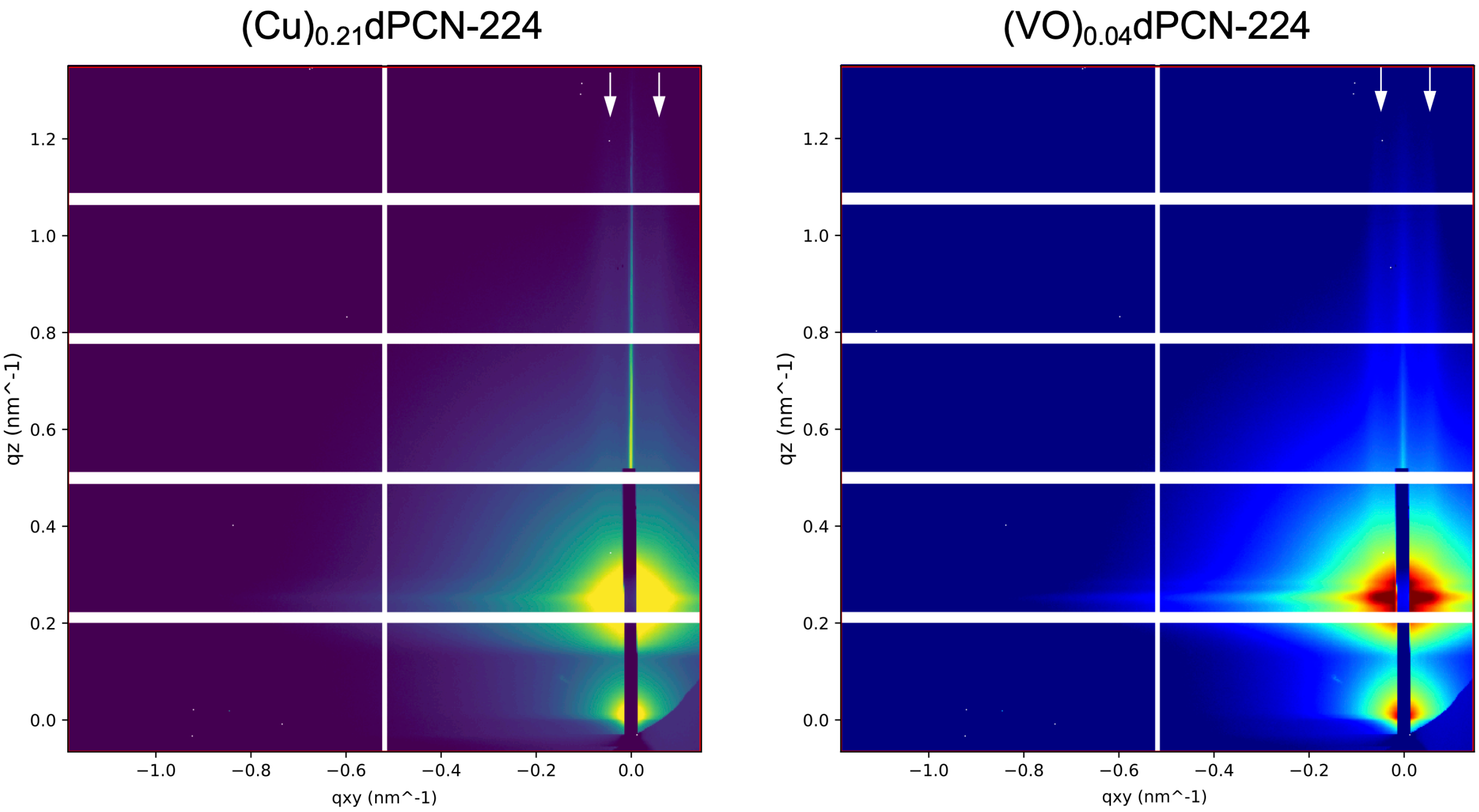

Figure S24. 2D plot the GISAXS data for $(Cu)_{0.21}$dPCN-224 (left) and $(VO)_{0.04}$dPCN-224 NPs (right) films transferred on Si by inverse-Langmuir-Schaefer. White arrows indicate Bragg rods resulting from particle-particle distances in the films.

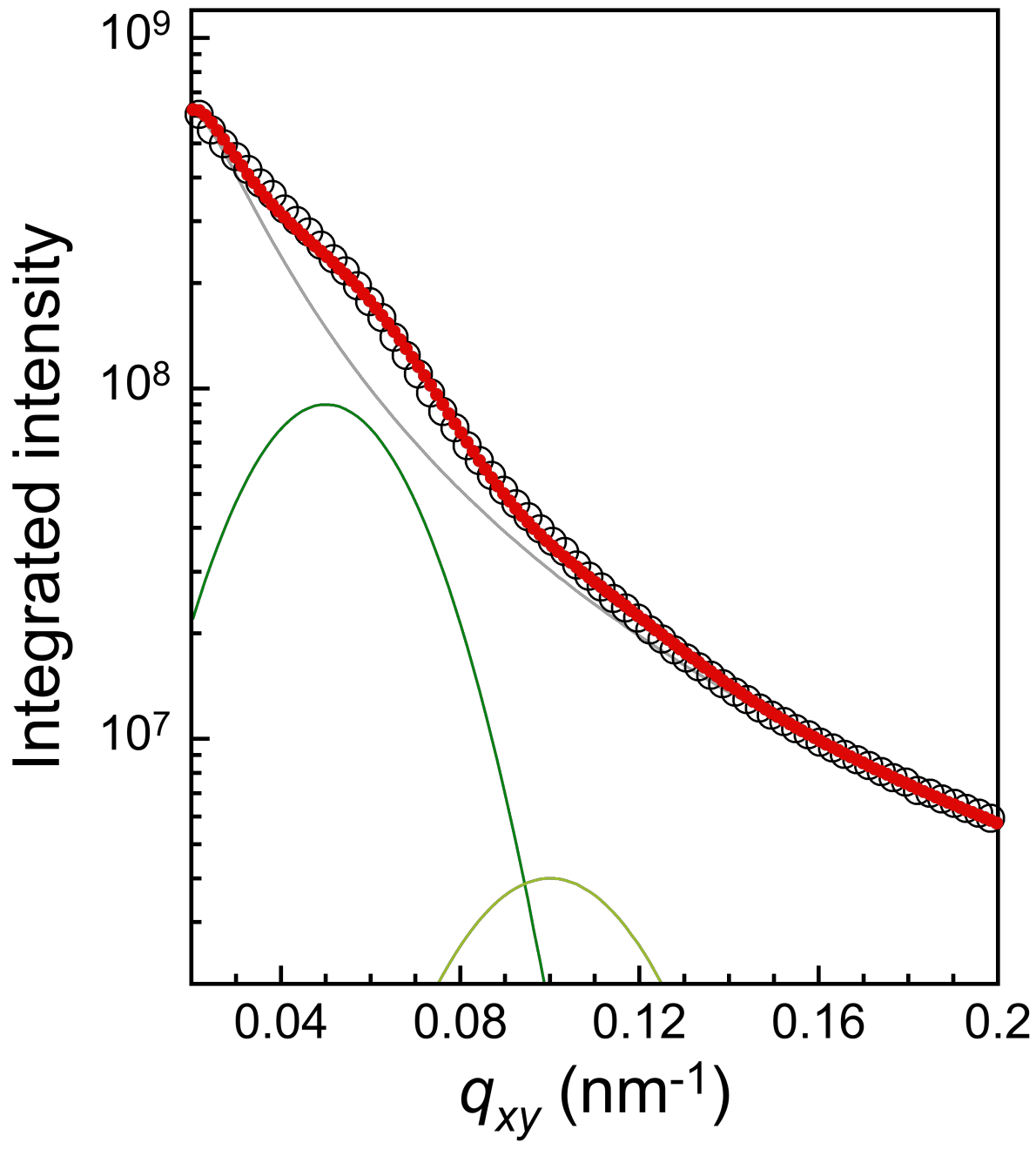

Figure S25. GISAXS data for a $(Cu)_{0.21}$dPCN-224 NPs film transferred on Si by inverse-Langmuir-Schaefer. The thick red line is the best-fit of the data by a multiple power-law background (main component $q^{-2.40}$, grey line) and two Gaussian peak(s) with maxima at q = 0.050(2)/0.100(3) nm^{-1} and width of 0.025(2)/0.030(3) nm^{-1} (green lines).

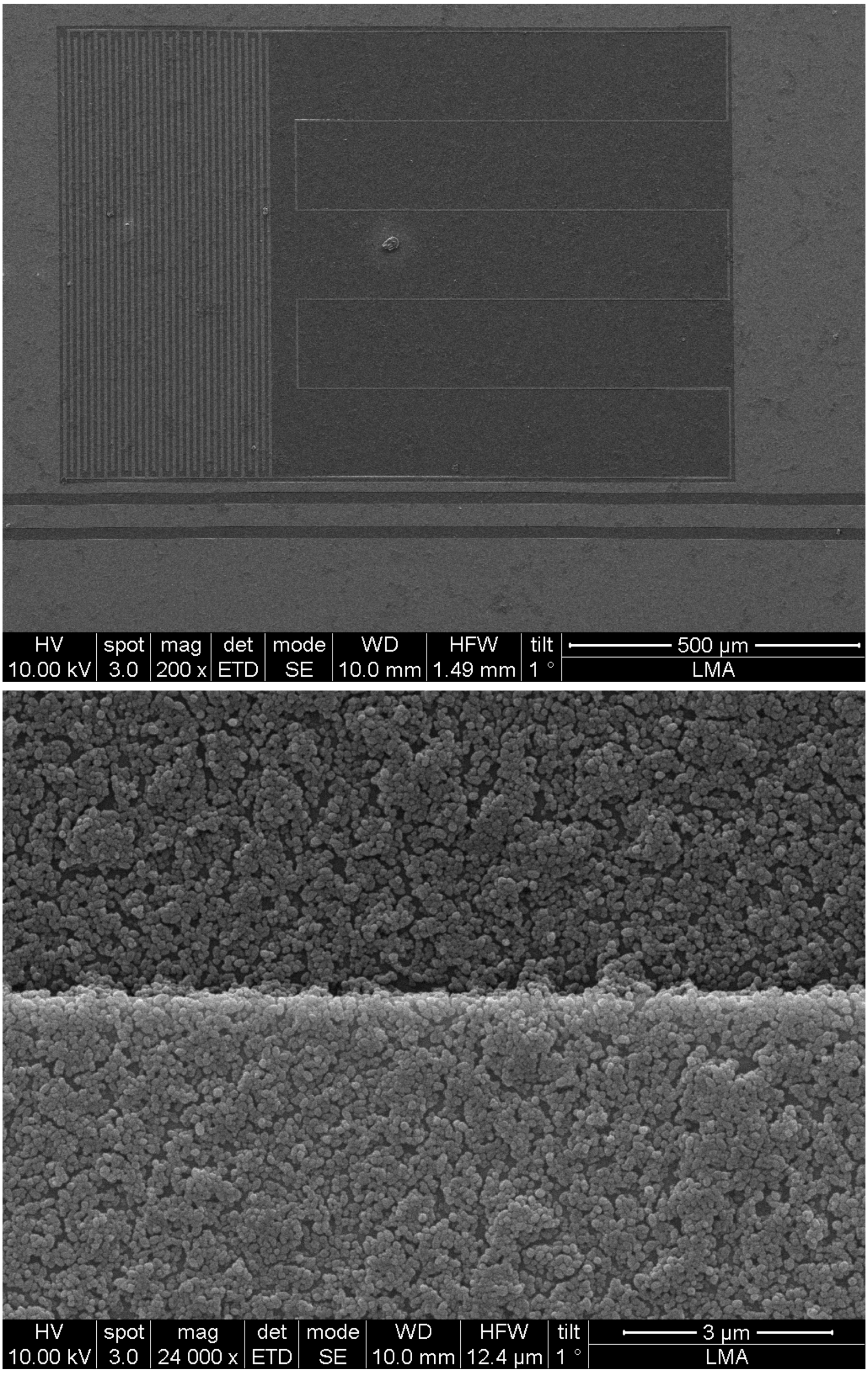

Figure S26. SEM images of $(Cu)_{0.21}$dPCN-224 NPs film transferred onto a chip with superconducting Nb LERs at 25 mN/m, showing a different resonator as that in Figure 5.

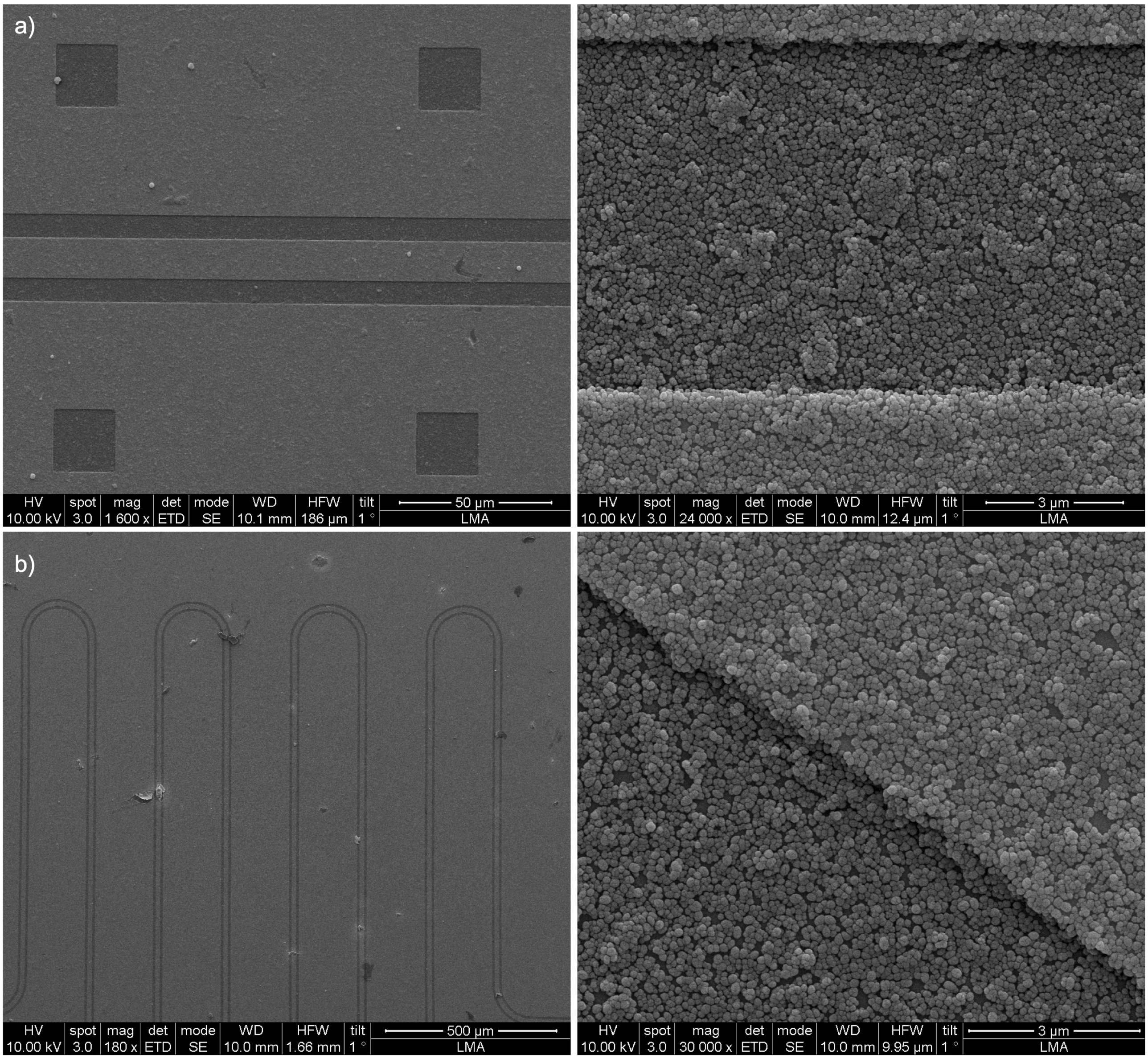

Figure S27. Representative SEM images of $(VO)_{0.04}$dPCN-224 NPs film transferred at 25 mN/m onto a chip with a coplanar waveguide Nb resonator, showing a) the superconducting line at the center of the chip and b) typical curved structure of the CPW, both at two magnifications.
